# CT habitat and peritumoral radiomics for predicting progression after repeat TACE in hepatocellular carcinoma: a multicenter study

**DOI:** 10.3389/fphar.2026.1942002

**Published:** 2026-10-14

**Authors:** Fei Wang, Yue Hu, Zhong-Xing Shi, Xin-Yu He, Yu-Hang Sui, Fu-Kai Wen, Jia-Shan Zhan, Ning-Jing Yang, Xue-Jing Han, Jia-Hui Wang, Jia-Yi Yang, Wen Lv, Hong-Fei Liu, Li-Ming Cui

**Affiliations:** 1 Department of Interventional Radiology, The Second Affiliated Hospital of Harbin Medical University, Harbin, Heilongjiang, China; 2 Department of Central Sterile Supply, The Second Affiliated Hospital of Harbin Medical University, Harbin, Heilongjiang, China; 3 Department of Hepatobiliary Surgery, The First Affiliated Hospital of Harbin Medical University, Harbin, Heilongjiang, China; 4 Department of Radiology, The First Affiliated Hospital of Wenzhou Medical University, Wenzhou, Zhejiang, China

**Keywords:** artificial intelligence, habitat radiomics, hepatocellular carcinoma, peritumoral radiomics, transarterial chemoembolization

## Abstract

**Background:**

This study developed and validated an machine-learning-based radiomics model integrating tumor habitat radiomics, peritumoral radiomics, and clinical features to predict progression-free survival (PFS) following repeat transarterial chemoembolization (TACE) in patients with hepatocellular carcinoma (HCC).

**Methods:**

This retrospective multicenter study included 270 patients with HCC. Patients from Center 1 were assigned to training (n = 94) and internal validation (n = 41) cohorts, while Centers 2 (n = 82) and 3 (n = 53) served as external validation cohorts. Tumor habitats and 5-mm peritumoral regions were generated from contrast-enhanced CT images. Radiomics features were selected using univariate Cox and LASSO-Cox regression. A fusion model and HPC risk score were constructed by integrating selected radiomics signatures and clinical variables. Performance was assessed using time-dependent ROC, C-index, calibration, decision curve analysis, and Kaplan–Meier analyses.

**Results:**

The optimal habitat cluster number was K = 2. Eleven habitat features, three peritumoral features, and alpha-fetoprotein were retained in the fusion model. The model achieved AUCs of 0.923, 0.921, and 0.897 for 3-, 6-, and 9-month progression-free survival in the training cohort, and 0.932, 0.863, and 0.916 in the internal validation cohort, respectively; all external AUCs exceeded 0.82. The fusion model showed higher C-index values than the clinical model across cohorts. High-risk patients had significantly shorter PFS than low-risk patients (all *P* < 0.001). Calibration and decision curve analyses demonstrated good agreement and clinical utility.

**Conclusion:**

The CT-based fusion model and HPC risk score effectively predicted progression risk in HCC.

## Introduction

Hepatocellular carcinoma (HCC) is one of the most common malignancies worldwide and is characterized by high incidence and poor prognosis (Filho et al., 2025). Owing to the lack of specific clinical manifestations at an early stage, most patients are already in the intermediate or advanced stages according to the Barcelona Clinic Liver Cancer (BCLC) staging system at the time of diagnosis, thereby losing the opportunity for curative treatment (Llovet et al., 2021). Transarterial chemoembolization (TACE), which selectively delivers chemotherapeutic agents and embolic materials into the tumor-feeding arteries to block the tumor blood supply, is an important treatment modality for intermediate- and advanced-stage HCC(3) (Heimbach et al., 2018).

In clinical practice, the decision to perform repeat TACE is usually based on follow-up imaging assessment, and some patients with a large tumor burden often require multiple TACE sessions to achieve satisfactory local control (Raoul et al., 2014; Zangos et al., 2007). However, repeated ineffective TACE not only fails to effectively control tumor progression but may also further impair liver function and adversely affect overall prognosis (Zhang et al., 2022). Therefore, establishing an effective assessment system to identify patients who cannot derive sustained benefit from repeat TACE and to adjust treatment strategies in a timely manner is of great clinical significance.

Previous studies have proposed retreatment assessment systems, such as the ART score and ABCR score, to predict survival benefit after sequential TACE (7, 8). In recent years, the application of radiomics in evaluating the efficacy of TACE in patients with HCC has received increasing attention, as it can provide noninvasive quantitative information to assist in treatment response assessment and prognostic prediction. However, most studies (Dai et al., 2025; Wang Y. et al., 2024; Dai et al., 2023) have primarily extracted features from the whole-tumor region, lacking explicit characterization of intratumoral spatial heterogeneity and the peritumoral microenvironment, and have mostly used overall survival (OS) as the endpoint, which makes it difficult to promptly reflect the actual tumor-control effect of TACE. In recent years, habitat-based radiomics has provided a new research strategy for characterizing intratumoral heterogeneity; by partitioning tumors into distinct imaging phenotypic regions, it can more precisely reflect the complexity of the tumor microenvironment (Cao et al., 2026; Huang et al., 2025). Meanwhile, the peritumoral region is also considered to contain important information related to tumor aggressiveness and prognosis (Huang and Qian, 2024; Li et al., 2022). However, studies integrating habitat features with peritumoral information for early progression prediction in patients undergoing repeat TACE remain lacking.

Therefore, in this study, we developed a fusion predictive model based on contrast-enhanced CT by integrating tumor habitat features, peritumoral radiomics features, and clinical variables, with progression-free survival (PFS) after the second TACE session as the prediction endpoint. Compared with overall survival, PFS can reflect tumor progression earlier and is more closely aligned with the clinical decision-making need to determine whether TACE should be continued. Furthermore, this study constructed a risk score and nomogram to enable individualized assessment of progression risk and provide supportive evidence for clinical treatment decision-making.

## Methods

### Patients

This retrospective multicenter study included patients with HCC from the Second Affiliated Hospital of Harbin Medical University (Center 1), the First Affiliated Hospital of Harbin Medical University (Center 2), and the First Affiliated Hospital of Wenzhou Medical University (Center 3). This study was approved by the institutional review board (approval number: YJSKY2025-087), and the requirement for informed consent was waived. Center 1 included 135 patients with HCC from January 2019 to December 2024, Center 2 included 82 patients during the same period, and Center 3 included 53 patients from January 2018 to December 2024. The inclusion criteria were as follows: a) HCC confirmed by histological or imaging criteria; b) receipt of initial and sequential second TACE treatments; c) completion of contrast-enhanced CT before the initial TACE; d) complete clinical data; e) follow-up imaging after the initial TACE showing viable intrahepatic lesions amenable to repeat embolization; and f) liver function and performance status considered sufficient to tolerate repeat TACE based on clinical assessment. The exclusion criteria were as follows: a) a history of other malignancies; b) prior invasive treatment before the initial TACE, such as radiofrequency ablation, surgery, or radiotherapy; and c) suboptimal CT image quality. Patients from Center 1 were randomly divided into a training cohort and an internal validation cohort at a ratio of 7:3, while Centers 2 and 3 served as independent external test cohorts. The patient screening process is shown in Figure 1.

**FIGURE 1:**
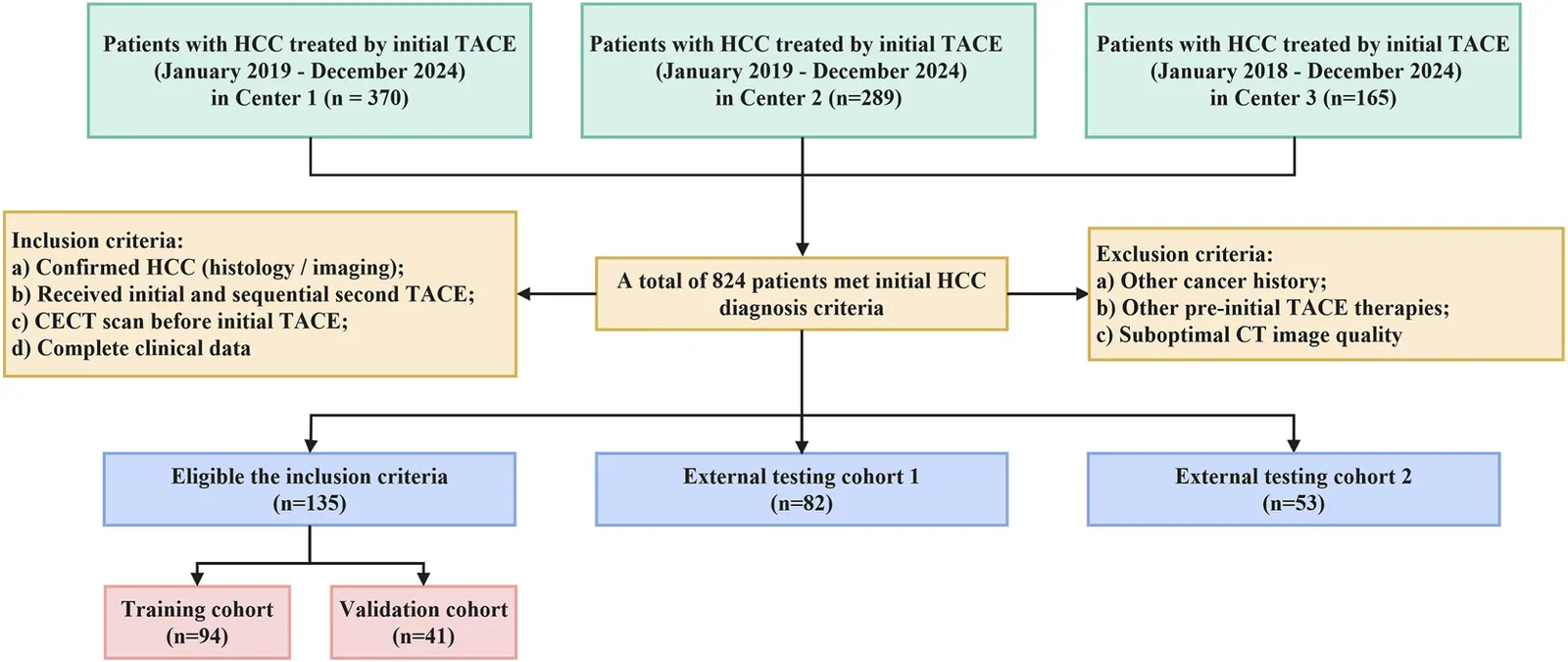
Flowchart of patient enrollment. CECT, Contrast-enhanced computed tomography; CT, Computed tomography; HCC, Hepatocellular carcinoma; TACE, Transarterial chemoembolization.

### Treatment procedure and follow-up

All patients underwent TACE *via* the femoral artery approach. Under digital subtraction angiography (DSA) guidance, a microcatheter was superselectively advanced into the tumor-feeding arteries, followed by injection of a chemotherapeutic emulsion using iodized oil as the carrier, mainly containing epirubicin or platinum-based agents, with the dose adjusted according to body surface area and liver function status. According to tumor vascularity and lesion characteristics, supplementary embolization with gelatin sponge particles or embolic microspheres was performed when necessary until a satisfactory embolization endpoint was achieved. Contrast-enhanced CT was performed within 4–8 weeks after the procedure, and treatment response was classified as complete response (CR), partial response (PR), stable disease (SD), or progressive disease (PD) according to the modified Response Evaluation Criteria in Solid Tumors (mRECIST) (Lencioni and Llovet, 2010). Imaging response was independently assessed by two radiologists with 10 years of experience, and disagreements were resolved by a senior radiologist with more than 20 years of experience. For patients whose follow-up imaging showed viable intrahepatic lesions amenable to repeat embolization and whose liver function and performance status were considered sufficient to tolerate repeat treatment, the clinical treatment team determined whether to perform a second TACE based on a comprehensive assessment of the response to the initial TACE and the anticipated benefits and safety of repeat treatment. During the study period, no uniform guideline-based definition of TACE refractoriness or failure was applied across the three participating centers. Because this study focused on the second TACE procedure, criteria for TACE refractoriness or failure requiring an insufficient response after two consecutive adequately performed TACE procedures could not be fully assessed before the second TACE. Therefore, the decision to perform the second TACE was based primarily on the comprehensive clinical assessment described above rather than on predefined and uniformly applied criteria for TACE refractoriness or failure. Thereafter, patients underwent contrast-enhanced CT or MRI follow-up every 8–12 weeks, with follow-up ending in December 2025; all patients completed follow-up, and no cases were lost to follow-up. By the end of follow-up, PFS events were observed in most patients, and the proportion of censored data was low. The primary endpoint of this study was PFS, defined as the time interval from the second TACE treatment to radiological progression or death.

### CT imaging acquisition and image segmentation

All patients underwent preoperative contrast-enhanced CT examinations. To reduce the impact of differences in scanning parameters, all images were uniformly resampled to an isotropic voxel spacing of 1 × 1 × 1 mm^3^. Subsequently, arterial phase (AP) images were spatially registered to portal venous phase (PVP) images using a Python-based platform, thereby ensuring spatial consistency of imaging information across different phases. Using PVP images as the reference, two physicians with experience in hepatic interventional imaging manually delineated the tumor region slice by slice with 3D Slicer software to construct a three-dimensional volume of interest (VOI) covering the entire lesion. A subset of cases was randomly selected for repeated segmentation, and segmentation reproducibility was assessed using the intraclass correlation coefficient (ICC). All segmentation results were reviewed and, when necessary, corrected by a senior radiologist with more than 20 years of experience.

The CT scanners and acquisition protocols varied to some extent across the three participating centers, including differences in tube voltage, tube current, slice thickness, contrast agent administration protocols, and scan timing for the contrast-enhanced phases. The main CT acquisition parameters for each center are summarized in Supplementary Table S1. To reduce image variability caused by differences in scanners and acquisition protocols, all images were preprocessed and resampled to a uniform voxel size before radiomics feature extraction.

### Peritumoral and habitat region generation

Three-dimensional morphological dilation of the tumor boundary was performed using Python (version 3.9.25), extending outward by 5 mm, and the original tumor region was subtracted to obtain the peritumoral region. To reduce the influence of adjacent nonhepatic tissues on feature extraction, a liver mask was generated using the deep learning–based automatic segmentation model nnU-Net, and the peritumoral region was constrained within the liver parenchyma. nnU-Net can automatically optimize the network architecture and preprocessing workflow according to data characteristics and has demonstrated favorable robustness and generalizability in various medical image segmentation tasks (Isensee et al., 2021). All segmentation results were manually reviewed, and standardized 5-mm peritumoral regions were ultimately obtained for subsequent radiomics feature extraction.

To characterize intratumoral spatial heterogeneity, habitat subregions were further constructed at the voxel level in this study. First, gray-level clipping was applied to the PVP images and the registered AP images within a range of −100 to 250 HU. Subsequently, the local mean and standard deviation were calculated within a 3 × 3 × 3 voxel neighborhood, and a six-dimensional voxel feature vector was constructed, including the gray values, local means, and standard deviations from both the PVP and AP images. After within-case standardization, the K-means clustering algorithm, implemented in scikit-learn, was used to perform unsupervised classification of intratumoral voxels. The number of clusters was evaluated within the prespecified range of K = 2–6, with the silhouette coefficient serving as the primary quantitative evaluation metric. The final K value was also determined by jointly considering the spatial distribution of the habitat subregions and their biological interpretability. Based on these considerations, K = 2 was selected for subsequent analyses, dividing the tumor into relatively hypoenhancing and hyperenhancing subregions. The Calinski–Harabasz index and Davies–Bouldin index were not prespecified as cluster-selection criteria in this study. According to the mean CT value in the portal venous phase, the hypoenhanced subregion was labeled as Habitat 1, whereas the hyperenhanced subregion was labeled as Habitat 2. No connected-component processing or removal of spatially isolated regions was performed after clustering. All voxels assigned to the same cluster were retained as a single habitat mask for radiomics feature extraction, regardless of whether they formed spatially disconnected regions.

### Radiomics feature extraction and selection

According to the guidelines of the Image Biomarker Standardisation Initiative (IBSI), radiomics features were extracted using PyRadiomics (version 3.0.1) in this study (Zwanenburg et al., 2020; Van Griethuysen et al., 2017). These features mainly included geometric features, first-order intensity features, and texture features, such as gray-level co-occurrence matrix (GLCM), gray-level run-length matrix (GLRLM), gray-level size-zone matrix (GLSZM), and neighboring gray-tone difference matrix (NGTDM) features. To ensure feature stability, segmentation reproducibility was assessed using the ICC, and ICC >0.80 was used as the selection threshold to exclude features with poor reproducibility. Because habitat subregions were constructed based on unsupervised clustering, which involves randomness and non-one-to-one correspondence between regions, ICC assessment was not performed for habitat features.

All radiomics features were Z-score standardized within the training cohort, and the same parameters were applied to the validation and external cohorts. Subsequently, features with no variation were removed, missing values were imputed using the median values from the training cohort, and redundant features were further excluded through low-variance filtering and Spearman correlation analysis (|r| > 0.70). The remaining features were further entered into univariate Cox regression analysis to evaluate their association with PFS. To strictly control the false-positive rate, a threshold of *P* < 0.001 was used for feature selection in the univariate Cox regression analysis. On this basis, LASSO-Cox regression was further applied for feature selection, with the optimal penalty parameter determined through five-fold cross-validation. Features with nonzero regression coefficients were ultimately retained for subsequent model construction. All preprocessing and feature selection procedures were performed exclusively within the training cohort, and the same parameters were applied to the validation and external cohorts to avoid information leakage.

### Habitat and peritumoral models

In this study, radiomics models were constructed based on habitat regions and peritumoral regions, respectively, to evaluate the effects of intratumoral heterogeneity and the surrounding microenvironment on patient prognosis. The habitat model generated an overall habitat signature by integrating radiomics features from each subregion, whereas the peritumoral model was based on features extracted from the 5-mm region extending outward from the tumor boundary. After feature selection, the selected features were used to construct Cox proportional hazards models, and individual risk scores were calculated for subsequent prognostic assessment and risk stratification analysis.

### Clinical model

Baseline clinical and CT imaging characteristics were collected, including sex, age, viral hepatitis status, portal vein tumor thrombus, extrahepatic metastasis, BCLC stage, Child–Pugh class, and serological indicators such as AFP, albumin, NLR, PLR, and SII. AFP was included in the analysis as a continuous variable using the original measured values without logarithmic transformation. First, univariate Cox regression analysis was performed to screen potential predictors associated with PFS. Subsequently, variables with statistical significance were entered into multivariate Cox regression analysis to identify independent prognostic factors.

### Fusion model

Habitat radiomics features, peritumoral radiomics features, and clinical variables were integrated to construct the fusion model. The habitat score, peritumoral score, and AFP were entered as covariates into multivariate Cox regression analysis to evaluate their independent contributions. Based on the final multivariate Cox model, an integrated risk score was calculated as a weighted linear combination of regression coefficients and was defined as the HPC risk score, where HPC represents habitat, peritumoral, and clinical features. A nomogram was subsequently constructed to predict PFS probabilities at different time points after repeat TACE. The workflow for predictive model development is shown in Figure 2.

**FIGURE 2:**
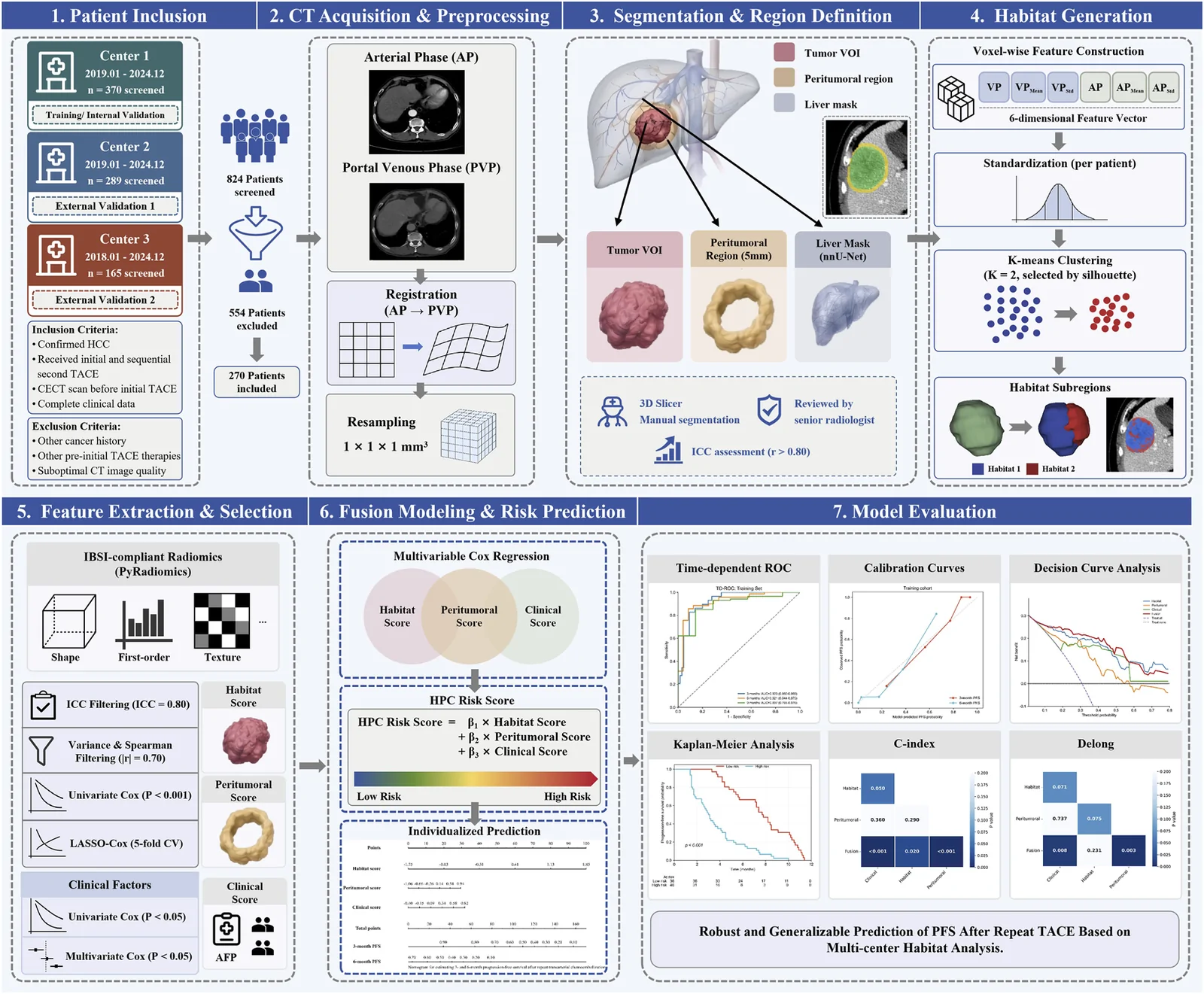
Flowchart of patient enrollment. AFP, alpha-fetoprotein; AP, arterial phase; APmean, local mean of arterial phase; APstd, local standard deviation of arterial phase; AUC, area under the curve; CT, computed tomography; DCA, decision curve analysis; DeLong, DeLong test; HCC, hepatocellular carcinoma; HPC, habitat, peritumoral, and clinical features; ICC, intraclass correlation coefficient; IBSI, Image Biomarker Standardisation Initiative; PFS, progression-free survival; PVP, portal venous phase; ROC, receiver operating characteristic; TACE, transarterial chemoembolization; VOI, volume of interest; VP, venous phase; VPmean, local mean of venous phase; VPstd, local standard deviation of venous phase.

### Statistical analysis and model evaluation

The normality of continuous variables was assessed using the Shapiro–Wilk test. Normally distributed continuous variables were expressed as the mean ± standard deviation and compared across multiple cohorts using one-way analysis of variance. Non-normally distributed continuous variables were expressed as the median and interquartile range and compared across groups using the Kruskal–Wallis test. Categorical variables were expressed as frequencies and percentages and compared using the chi-square test or Fisher’s exact test, as appropriate. The cut-off value was determined based on the median risk score in the training cohort, and patients were stratified into high-risk and low-risk groups. Differences between groups were evaluated using Kaplan–Meier survival curves and the log-rank test. Model discrimination was assessed using the C-index and time-dependent receiver operating characteristic (time-dependent ROC) curves, and the areas under the curve (AUCs) for 3-, 6-, and 9-month PFS were calculated. Model calibration was evaluated using calibration curves and the Brier score. Calibration curves were used to illustrate the agreement between the model-predicted probabilities and the observed outcomes, whereas the Brier score was used to quantitatively assess the overall prediction error at different time points. Considering that clinical decision-making regarding repeat TACE is usually focused on the early post-treatment period, and that most disease progression events in this study occurred within the first 6 months, whereas the number of cases available for stable evaluation at 9 months was relatively limited, calibration curves, Brier scores, and decision curve analysis were mainly focused on the 3- and 6-month time points. All statistical tests were two-sided, and a two-sided *P* < 0.05 was considered statistically significant.

### Reporting guidelines

This study and its predictive models were reported with reference to the TRIPOD statement and the CLEAR checklist for medical imaging artificial intelligence studies. The completed TRIPOD and CLEAR checklists are provided in the supplementary materials.

## Results

### Patients and clinical characteristics

Table 1 summarizes the baseline clinical characteristics of the patients. The distributions of most variables were balanced across cohorts, with no statistically significant differences observed (*P* > 0.05). A mild difference in age distribution was observed among the five cohorts (*P* = 0.046), mainly reflected by a slightly higher median age in external validation cohort 2 than in the other cohorts. Overall, the baseline clinical characteristics were relatively well balanced across cohorts.

**TABLE 1:** Baseline characteristics of patients with hepatocellular carcinoma across cohorts.

Characteristic	Training cohort	Internal validation cohort	External validation cohort 1	External validation cohort 2	External combined cohort	*P*
	(*n* = 94)	(*n* = 41)	(*n* = 82)	(*n* = 53)	(*n* = 135)	​
Age (y)	57.00 (49.25, 64.75)	56.00 (47.00, 64.00)	56.50 (47.25, 67.00)	60.00 (55.00, 69.00)	59.00 (51.00, 68.00)	0.046
Sex	​	​	​	​	​	0.548
M	83 (88.3%)	34 (82.9%)	66 (80.5%)	45 (84.9%)	111 (82.2%)	​
F	11 (11.7%)	7 (17.1%)	16 (19.5%)	8 (15.1%)	24 (17.8%)	​
Viral hepatitis	73 (77.7%)	33 (80.5%)	69 (84.1%)	37 (69.8%)	106 (78.5%)	0.255
PVTT	29 (30.9%)	15 (36.6%)	15 (18.3%)	12 (22.6%)	27 (20.0%)	0.097
Extrahepatic Spread	24 (25.5%)	10 (24.4%)	18 (22.0%)	7 (13.2%)	25 (18.5%)	0.359
BCLC stage	​	​	​	​	​	0.433
A	14 (14.9%)	11 (26.8%)	20 (24.4%)	11 (20.8%)	31 (23.0%)	​
B	48 (51.1%)	19 (46.3%)	40 (48.8%)	31 (58.5%)	71 (52.6%)	​
C	32 (34.0%)	11 (26.8%)	22 (26.8%)	11 (20.8%)	33 (24.4%)	​
Child-Pugh class base	​	​	​	​	​	0.292
A	75 (79.8%)	34 (82.9%)	74 (90.2%)	45 (84.9%)	119 (88.1%)	​
B	19 (20.2%)	7 (17.1%)	8 (9.8%)	8 (15.1%)	16 (11.9%)	​
AFP	90.91 (11.99, 1300.00)	50.79 (9.97, 266.00)	10.65 (5.25, 583.63)	26.69 (5.63, 245.63)	21.21 (5.45, 577.87)	0.108
ALT	32.00 (22.00, 52.00)	33.00 (23.00, 49.00)	37.50 (25.00, 49.75)	29.00 (22.00, 43.00)	34.00 (23.00, 48.00)	0.629
AST	41.00 (31.00, 69.50)	47.00 (25.00, 66.00)	42.50 (29.50, 69.25)	40.00 (29.00, 58.00)	41.00 (29.00, 68.50)	0.956
ALB	40.75 (38.05, 43.08)	40.70 (34.90, 43.90)	40.20 (36.30, 43.70)	40.00 (36.60, 42.70)	40.20 (36.40, 43.20)	0.89
DBIL	6.90 (5.60, 9.28)	5.90 (4.90, 7.90)	6.25 (4.83, 8.43)	6.50 (5.60, 9.10)	6.30 (5.00, 8.55)	0.112
IBIL	9.10 (6.83, 13.47)	8.10 (6.60, 10.00)	8.90 (6.40, 11.38)	9.60 (6.80, 12.70)	9.20 (6.55, 11.60)	0.216
PT	12.50 (11.70, 13.10)	12.40 (11.80, 13.20)	12.45 (11.93, 13.50)	12.60 (11.80, 13.70)	12.50 (11.90, 13.60)	0.368
INR	1.12 (1.04, 1.19)	1.12 (1.08, 1.20)	1.14 (1.09, 1.21)	1.15 (1.07, 1.24)	1.14 (1.09, 1.22)	0.06
TT	14.90 (14.15, 16.20)	15.10 (14.00, 15.90)	15.50 (14.53, 17.20)	15.20 (14.00, 16.40)	15.40 (14.40, 17.20)	0.162
APTT	35.60 (33.82, 38.55)	35.90 (33.50, 40.60)	36.90 (33.78, 40.48)	34.80 (31.30, 37.90)	36.20 (33.25, 39.15)	0.071
RBC	4.44 (4.10, 4.77)	4.55 (3.87, 4.84)	4.28 (3.88, 4.72)	4.31 (3.89, 4.72)	4.30 (3.88, 4.72)	0.38
HB	140.00 (128.25, 147.75)	142.00 (128.00, 157.00)	133.00 (122.00, 150.00)	137.00 (121.00, 148.00)	135.00 (121.50, 150.00)	0.174
NLR	2.60 (1.87, 3.81)	2.96 (1.92, 4.00)	2.62 (1.88, 3.80)	3.04 (1.96, 4.15)	2.67 (1.90, 3.97)	0.638
MLR	0.46 (0.34, 0.58)	0.48 (0.38, 0.62)	0.46 (0.29, 0.54)	0.44 (0.25, 0.54)	0.46 (0.27, 0.54)	0.088
PLR	104.69 (71.69, 159.48)	104.23 (70.26, 163.16)	119.08 (73.62, 161.41)	116.67 (71.74, 185.71)	117.02 (72.33, 175.23)	0.701
SII	429.25 (246.83, 726.34)	499.33 (247.17, 724.00)	372.80 (208.90, 692.15)	444.09 (199.95, 917.33)	385.94 (203.47, 776.13)	0.487

AFP, alpha-fetoprotein; ALB, albumin; ALT, alanine aminotransferase; APTT, activated partial thromboplastin time; AST, aspartate aminotransferase; BCLC, barcelona clinic liver cancer; DBIL, direct bilirubin; HB, hemoglobin; IBIL, indirect bilirubin; INR, international normalized ratio; MLR, monocyte-to-lymphocyte ratio; NLR, neutrophil-to-lymphocyte ratio; PLR, platelet-to-lymphocyte ratio; PT, prothrombin time; PVTT, portal vein tumor thrombus; RBC, red blood cell; SII, systemic immune-inflammation index; TT, thrombin time.

The results of univariate and multivariate Cox regression analyses are presented in Table 2. Univariate analysis showed that AFP and AST were significantly associated with patient PFS, with both *P* < 0.05. After further inclusion in multivariate Cox regression analysis, only AFP remained statistically significant (HR = 1.001, 95% CI: 1.001–1.001, *P* < 0.001), whereas AST did not show independent predictive value (*P* = 0.123). Therefore, AFP was retained as the final clinical predictor for subsequent fusion model construction.

**TABLE 2:** Univariate and multivariate Cox regression analyses of clinical variables in the training cohort.

Parameter	PFS	​	​	​
	Univariate	​	Multivariate	​
	HR (95% CI)	*P*	HR (95% CI)	*P*
Age	0.998 (0.976–1.020)	0.839	​	​
Sex_Male	1.601 (0.849–3.019)	0.146	​	​
Viral hepatitis	0.787 (0.472–1.311)	0.358	​	​
PVTT	1.425 (0.915–2.217)	0.117	​	​
Extrahepatic Spread	1.153 (0.722–1.842)	0.55	​	​
BCLC_B	1.034 (0.687–1.557)	0.872	​	​
BCLC_C	0.927 (0.604–1.424)	0.73	​	​
Child-Pugh_B	1.459 (0.885–2.404)	0.139	​	​
AFP	1.001 (1.001–1.001)	<0.001*	1.001 (1.001–1.001)	<0.001*
ALT	1.002 (0.995–1.008)	0.619	​	​
AST	1.003 (1.000–1.006)	0.036*	1.002 (0.999–1.006)	0.123
ALB	0.983 (0.947–1.021)	0.374	​	​
DBIL	1.014 (0.942–1.092)	0.706	​	​
IBIL	0.996 (0.953–1.042)	0.873	​	​
PT	0.950 (0.828–1.089)	0.461	​	​
INR	0.885 (0.185–4.247)	0.879	​	​
TT	0.921 (0.813–1.044)	0.197	​	​
APTT	1.027 (0.982–1.075)	0.241	​	​
RBC	1.325 (0.974–1.801)	0.073	​	​
HB	1.004 (0.994–1.013)	0.464	​	​
NLR	0.995 (0.978–1.013)	0.586	​	​
MLR	1.296 (0.741–2.264)	0.363	​	​
PLR	1.001 (0.999–1.004)	0.366	​	​
SII	1.000 (1.000–1.000)	0.938	​	​

AFP, alpha-fetoprotein; ALB, albumin; ALT, alanine aminotransferase; APTT, activated partial thromboplastin time; AST, aspartate aminotransferase; BCLC, barcelona clinic liver cancer; CI, confidence interval; DBIL, direct bilirubin; HB, hemoglobin; HR, hazard ratio; IBIL, indirect bilirubin; INR, international normalized ratio; MLR, monocyte-to-lymphocyte ratio; NLR, neutrophil-to-lymphocyte ratio; PFS, progression-free survival; PLR, platelet-to-lymphocyte ratio; PT, prothrombin time; PVTT, portal vein tumor thrombus; RBC, red blood cell; SII, systemic immune-inflammation index; TBIL, total bilirubin; TT, thrombin time.

### Subregion clustering and feature selection

Based on the silhouette coefficient, the optimal number of clusters was determined within the predefined range of K = 2–6. When K = 2, the silhouette coefficient reached its maximum value, with a mean of approximately 0.38 (Figure 3a). The tumor volume of interest was ultimately divided into two subregions, defined as Habitat 1 (H1) and Habitat 2 (H2), respectively (Figure 3b). Among them, H1 mainly exhibited relatively hypoenhanced characteristics, whereas H2 showed more hyperenhanced characteristics. In one representative case, H1 and H2 accounted for 62.5% and 37.5% of the total tumor volume, respectively, reflecting the complex spatial distribution of intratumoral heterogeneity. Figure 3c presents the habitat partitioning and the generated maps of selected features in the representative case.

**FIGURE 3:**
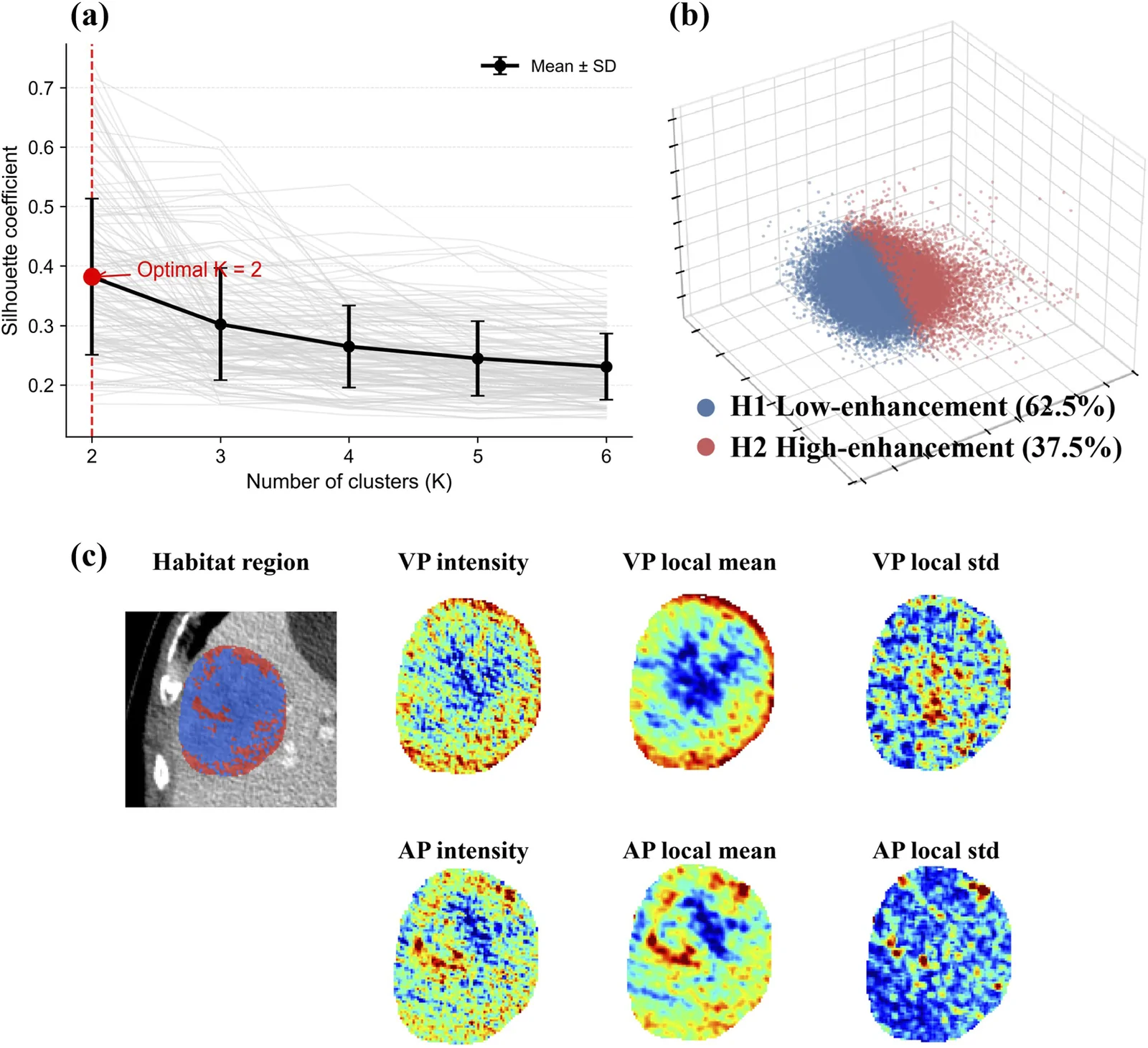
Habitat clustering results. **(a)** Line plot of silhouette coefficient scores. **(b)** Three-dimensional visualization of the clustering results. **(c)** Habitat generation and selected feature maps in a representative patient with hepatocellular carcinoma. AP, arterial phase; H1, Habitat 1; H2, Habitat 2; K, number of clusters; SD, standard deviation; VP, venous phase.

After feature selection using univariate Cox regression and LASSO-Cox regression, 11 radiomics features were ultimately retained to construct the habitat radiomics signature, and 3 radiomics features were retained to construct the peritumoral radiomics signature, with their corresponding regression coefficients shown in Supplementary Figure S1. The selected features included first-order statistical features and various higher-order texture features, and were derived from both original images and different image-transformed images.

### Construction of the fusion model and HPC risk score

Based on the clinical Cox regression results, AFP was retained as the clinical component of the fusion model. A fusion model was constructed by integrating the habitat score derived from 11 selected habitat features, the peritumoral score derived from 3 selected peritumoral features, and AFP, with the detailed coefficients and hazard ratios presented in Supplementary Table S2. The individual integrated risk score was defined as:

HPC risk score=0.294300×Habitat_score+0.371921×Peri_score+0.000205×AFP

Habitat_score and Peri_score represent the risk scores derived from the habitat and peritumoral models, respectively, whereas AFP represents the original serum AFP value measured in ng/mL. Because AFP was entered as a continuous variable in its original units, its coefficient of 0.000205 represents the change in the HPC risk score associated with a 1-ng/mL increase in AFP. Therefore, the smaller numerical coefficient should be interpreted in relation to the measurement scale of AFP and does not necessarily indicate a negligible contribution to the model. The distributions of the HPC risk score and its components differed significantly between the high- and low-risk groups (Figure 4). The cut-off value was determined based on the median HPC risk score in the training cohort (−0.076650), and patients were stratified into high-risk and low-risk groups. Patients in the high-risk group exhibited a higher risk of early progression, and this risk stratification was significantly validated across all validation cohorts, with all *P* < 0.05.

**FIGURE 4:**
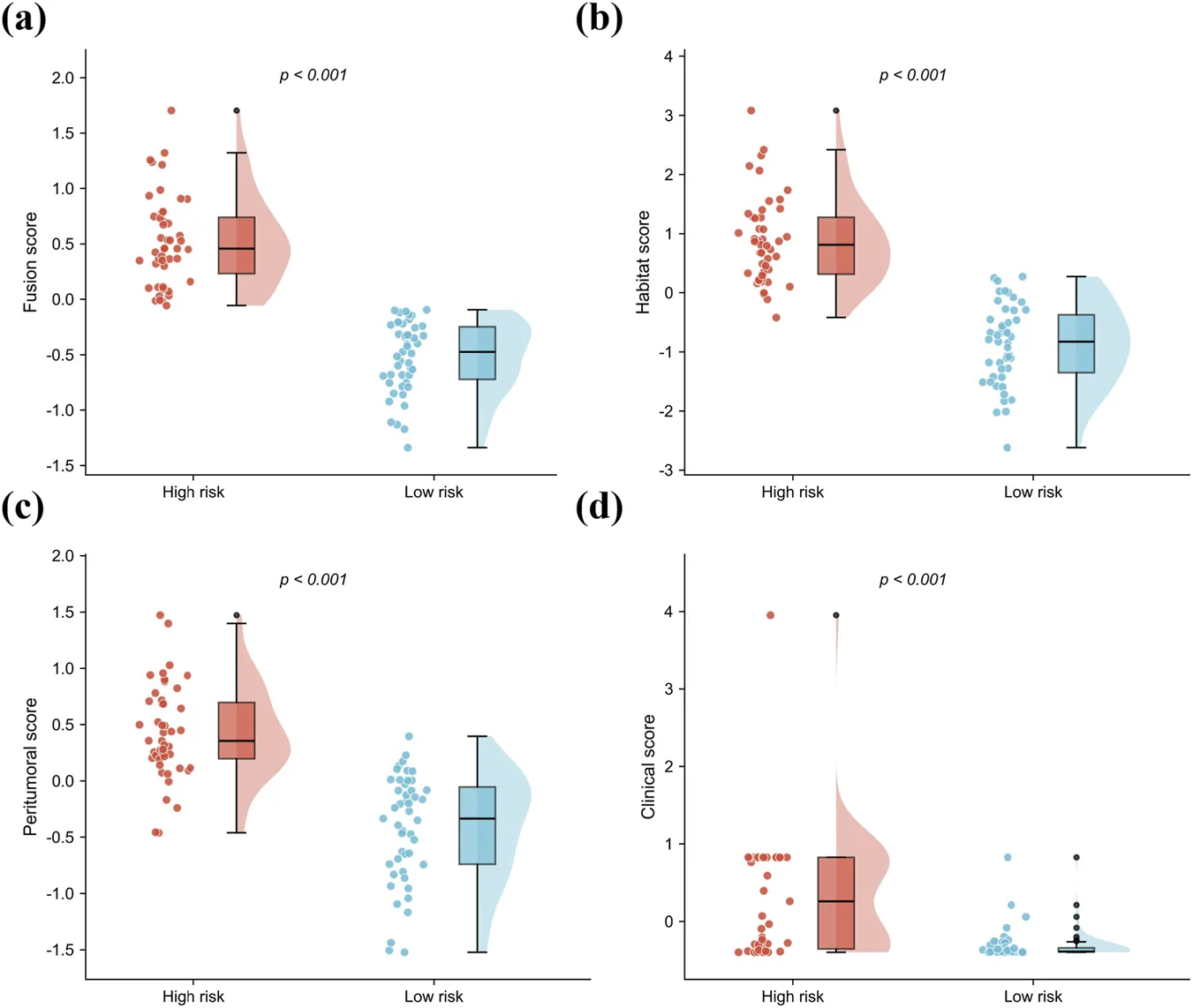
Comparison of the distributions of HPC risk score components between the high-risk and low-risk groups. **(a)** HPC risk score. **(b)** Habitat score. **(c)** Peritumoral score. **(d)** Clinical score. Risk scores derived from the fusion, habitat, peritumoral, and clinical models were all significantly higher in the high-risk group than in the low-risk group (all P < 0.001). Dots indicate individual patients, boxplots indicate the median and interquartile range, and shaded areas indicate score density.

#### Predictive performance of different models

Harrell’s C-index and time-dependent ROC curves were used to evaluate the predictive performance of each model, and the areas under the curve (AUCs) for 3-, 6-, and 9-month PFS were calculated. In the C-index analysis, the fusion model demonstrated good discriminative ability across all cohorts. However, its performance advantage over the individual models did not reach statistical significance in every cohort or in all pairwise comparisons. In the training cohort, the C-index of the fusion model was significantly higher than those of the clinical model, habitat model, and peritumoral model, with all *P* < 0.05 (Supplementary Figure S2). In the internal validation cohort and external validation cohorts, the C-index of the fusion model also remained consistently higher than that of the clinical model, with most *P* < 0.05 (Supplementary Figures S1-S6). In contrast, the differences between the fusion model and the habitat or peritumoral models did not reach statistical significance in most cohorts, suggesting that the single radiomics models already had strong independent discriminative ability. Time-dependent AUC analysis showed that the fusion model maintained favorable predictive performance at all time points (Table 3). In the training cohort, internal validation cohort, and external validation cohorts, the AUCs for 3-, 6-, and 9-month PFS were generally greater than 0.82 (Figure 5).

**TABLE 3:** Performance of different models for predicting progression-free survival in the training and test cohorts.

Cohort	Model	C-index (95% CI)	3-month AUC (95% CI)	6-month AUC (95% CI)	9-month AUC (95% CI)
Training cohort	Clinical	0.707 (0.639–0.769)	0.788 (0.660–0.890)	0.768 (0.657–0.868)	0.888 (0.795–0.961)
​	Habitat	0.772 (0.734–0.809)	0.896 (0.824–0.954)	0.895 (0.830–0.954)	0.819 (0.697–0.925)
​	Peritumoral	0.740 (0.685–0.788)	0.813 (0.723–0.896)	0.871 (0.774–0.957)	0.906 (0.809–0.982)
​	Fusion	0.815 (0.773–0.855)	0.923 (0.860–0.969)	0.921 (0.844–0.975)	0.897 (0.785–0.978)
Internal validation cohort	Clinical	0.686 (0.560–0.802)	0.647 (0.437–0.857)	0.757 (0.607–0.901)	0.784 (0.632–0.910)
​	Habitat	0.774 (0.720–0.825)	0.907 (0.812–0.986)	0.832 (0.698–0.948)	0.857 (0.726–0.971)
​	Peritumoral	0.747 (0.651–0.826)	0.895 (0.777–0.979)	0.777 (0.624–0.912)	0.861 (0.729–0.970)
​	Fusion	0.809 (0.751–0.861)	0.932 (0.845–0.990)	0.863 (0.744–0.968)	0.916 (0.816–0.992)
External validation cohort 1	Clinical	0.633 (0.557–0.702)	0.807 (0.664–0.932)	0.698 (0.583–0.807)	0.647 (0.529–0.758)
​	Habitat	0.762 (0.697–0.816)	0.875 (0.794–0.942)	0.839 (0.724–0.928)	0.872 (0.731–0.970)
​	Peritumoral	0.734 (0.669–0.793)	0.836 (0.734–0.928)	0.806 (0.696–0.899)	0.832 (0.731–0.922)
​	Fusion	0.792 (0.737–0.841)	0.920 (0.858–0.968)	0.872 (0.778–0.945)	0.892 (0.781–0.972)
External validation cohort 2	Clinical	0.642 (0.557–0.728)	0.723 (0.547–0.882)	0.709 (0.564–0.835)	0.699 (0.538–0.853)
​	Habitat	0.747 (0.654–0.823)	0.832 (0.665–0.959)	0.851 (0.730–0.950)	0.807 (0.659–0.922)
​	Peritumoral	0.693 (0.597–0.775)	0.768 (0.627–0.884)	0.800 (0.668–0.911)	0.729 (0.510–0.929)
​	Fusion	0.772 (0.681–0.847)	0.845 (0.687–0.965)	0.870 (0.764–0.957)	0.823 (0.675–0.935)
External combined cohort	Clinical	0.647 (0.590–0.700)	0.778 (0.670–0.872)	0.706 (0.613–0.796)	0.696 (0.600–0.782)
​	Habitat	0.758 (0.712–0.800)	0.853 (0.773–0.919)	0.850 (0.782–0.915)	0.849 (0.756–0.927)
​	Peritumoral	0.717 (0.665–0.765)	0.808 (0.719–0.881)	0.806 (0.728–0.881)	0.793 (0.676–0.891)
​	Fusion	0.785 (0.741–0.827)	0.887 (0.816–0.951)	0.874 (0.815–0.930)	0.865 (0.779–0.935)

AUC, area under the curve; CI, confidence interval; C-index, concordance index; PFS, progression-free survival.

**FIGURE 5:**
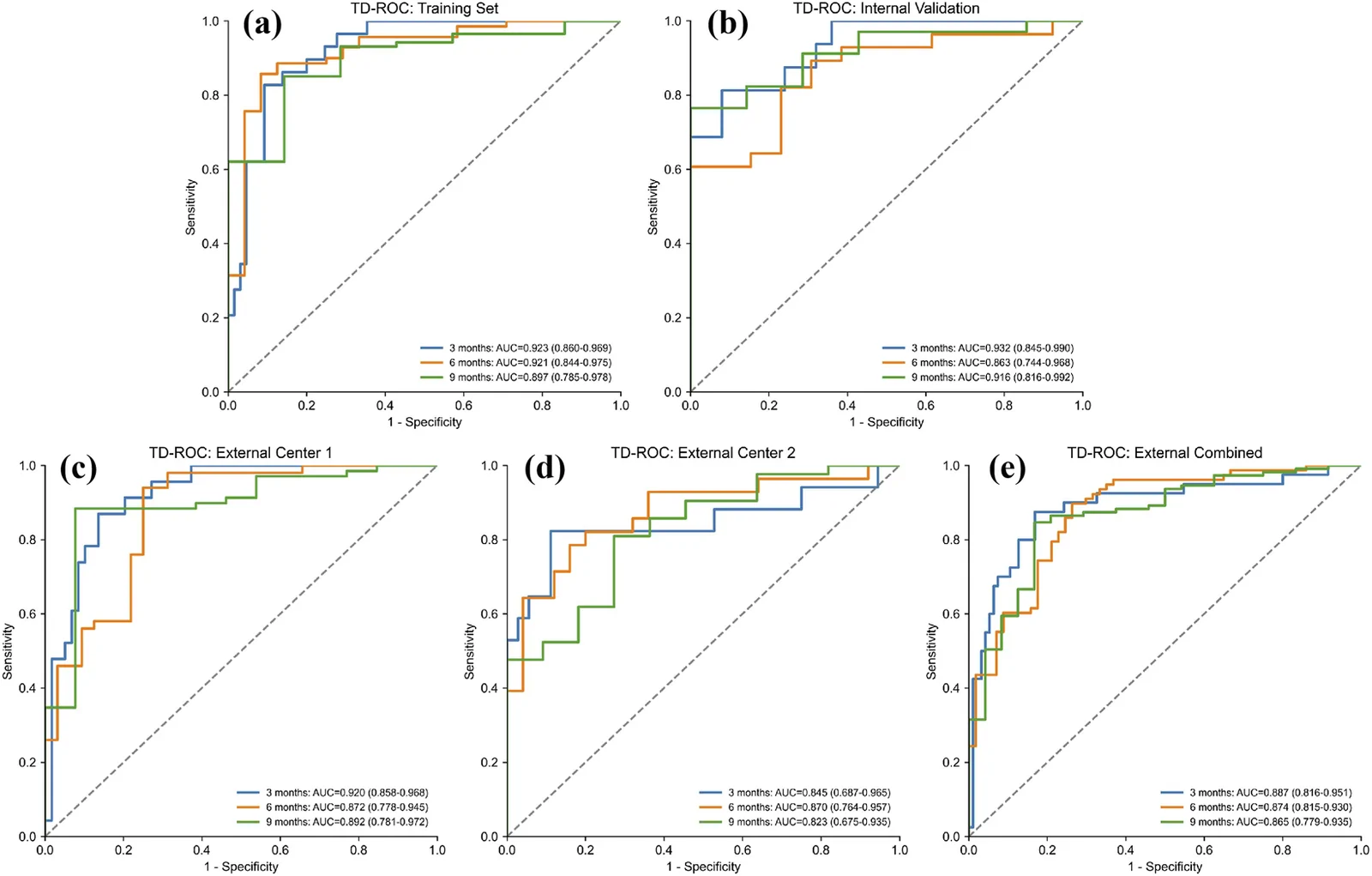
Time-dependent ROC curves of the fusion model. **(a–e)** Results in the training cohort, internal validation cohort, external test cohort 1, external test cohort 2, and external combined validation cohort, respectively. AUC, area under the curve; ROC, receiver operating characteristic.

DeLong tests were further used to compare AUC differences among different models at each time point (Supplementary Figures S2-S6). At the 3 months time point, the fusion model significantly outperformed the clinical model in the training cohort, internal validation cohort, external validation cohort 1, and external combined cohort, with all *P* < 0.05. In external validation cohort 2, however, the difference did not reach statistical significance (*P* = 0.168). Compared with the habitat model and peritumoral model, most comparisons involving the fusion model did not reach statistical significance, although the fusion model showed certain advantages over the peritumoral model in some cohorts (*P* < 0.05). At the 6-month time point, the fusion model remained significantly superior to the clinical model in the training cohort, external validation cohort 1, external validation cohort 2, and external combined cohort (*P* = 0.002, 0.006, 0.019, and <0.001, respectively). However, most comparisons between the fusion model and the habitat or peritumoral models still did not reach statistical significance. By 9 months, the differences among models were further attenuated. Although the fusion model still maintained numerically higher AUCs in most cohorts, it remained statistically superior to the clinical model only in external validation cohort 1 and the external combined cohort (*P* = 0.004 and 0.002, respectively).

Overall, the fusion model demonstrated superior predictive performance compared with the clinical model at multiple time points, with a more pronounced advantage in the early prediction stage, particularly at 3 months. Although its statistical superiority over single radiomics models was not consistently observed, the fusion model showed more stable overall performance and demonstrated favorable cross-center generalizability.

### Calibration and prediction error analysis

The calibration curves showed that the model-predicted probabilities were generally close to the observed outcomes, although some deviations were observed in certain external cohorts (Figure 6). The Brier scores provided a quantitative assessment of the model’s prediction error at the 3- and 6-month time points (Supplementary Figure S7). Taken together, these results indicate that the model demonstrated acceptable calibration across the different cohorts, although further validation in larger prospective cohorts is warranted.

**FIGURE 6:**
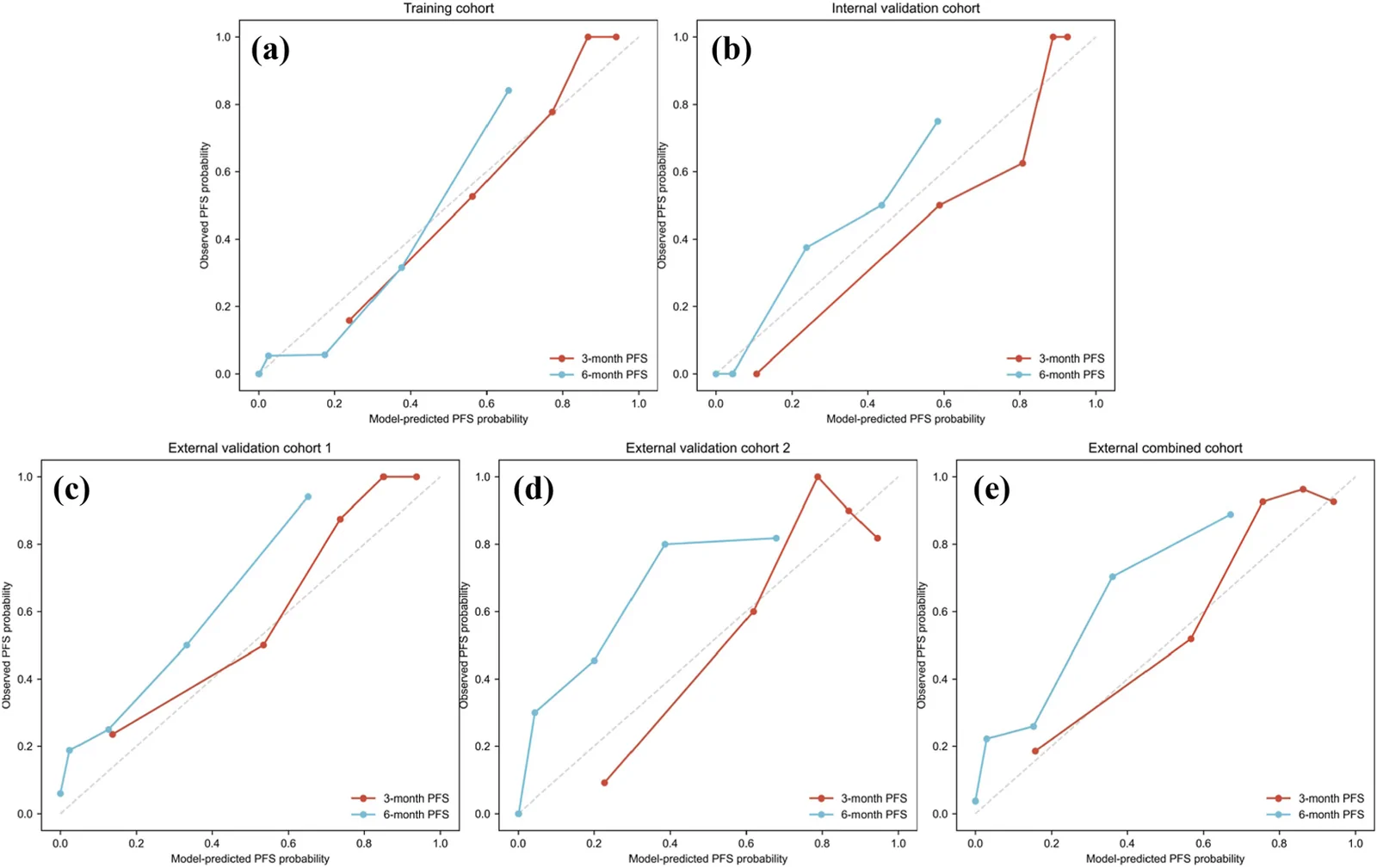
Calibration curves of the fusion model in each cohort. **(a–e)** Calibration curves for predicting 3- and 6-month progression-free survival in the training cohort, internal validation cohort, external test cohort 1, external test cohort 2, and external combined validation cohort, respectively. PFS, progression-free survival.

### Clinical utility analysis

Decision curve analysis showed that, for the 3- and 6-month predictions, the fusion model achieved the highest net benefit across most threshold probability ranges (Figure 7). In contrast, the single habitat model, peritumoral model, and clinical model showed lower net benefits. Across all cohorts, the fusion model outperformed the “treat-all” and “treat-none” strategies, indicating its potential clinical utility in decision-making, particularly within the intermediate threshold probability range of approximately 0.2–0.6, where it demonstrated higher net benefit.

**FIGURE 7:**
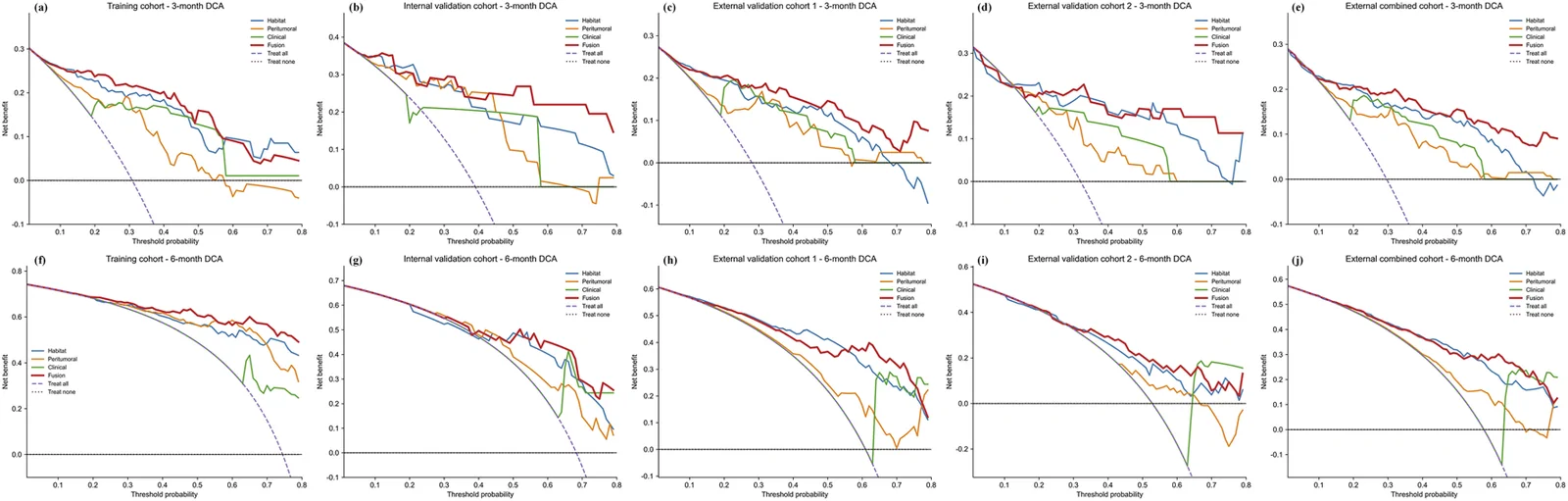
Decision curve analysis of the fusion model. **(a–e)** Decision curves for 3-month prediction in the training cohort, internal validation cohort, external test cohort 1, external test cohort 2, and external combined validation cohort, respectively. **(f–j)** Decision curves for 6-month prediction in the corresponding cohorts. DCA, decision curve analysis.

### Kaplan–Meier analysis of the HPC risk score

The cut-off value was determined based on the median HPC risk score in the training cohort (−0.076650), and patients were stratified into high-risk and low-risk groups. Kaplan–Meier curves and the log-rank test were used to evaluate the risk stratification ability of the model (Figure 8).

**FIGURE 8:**
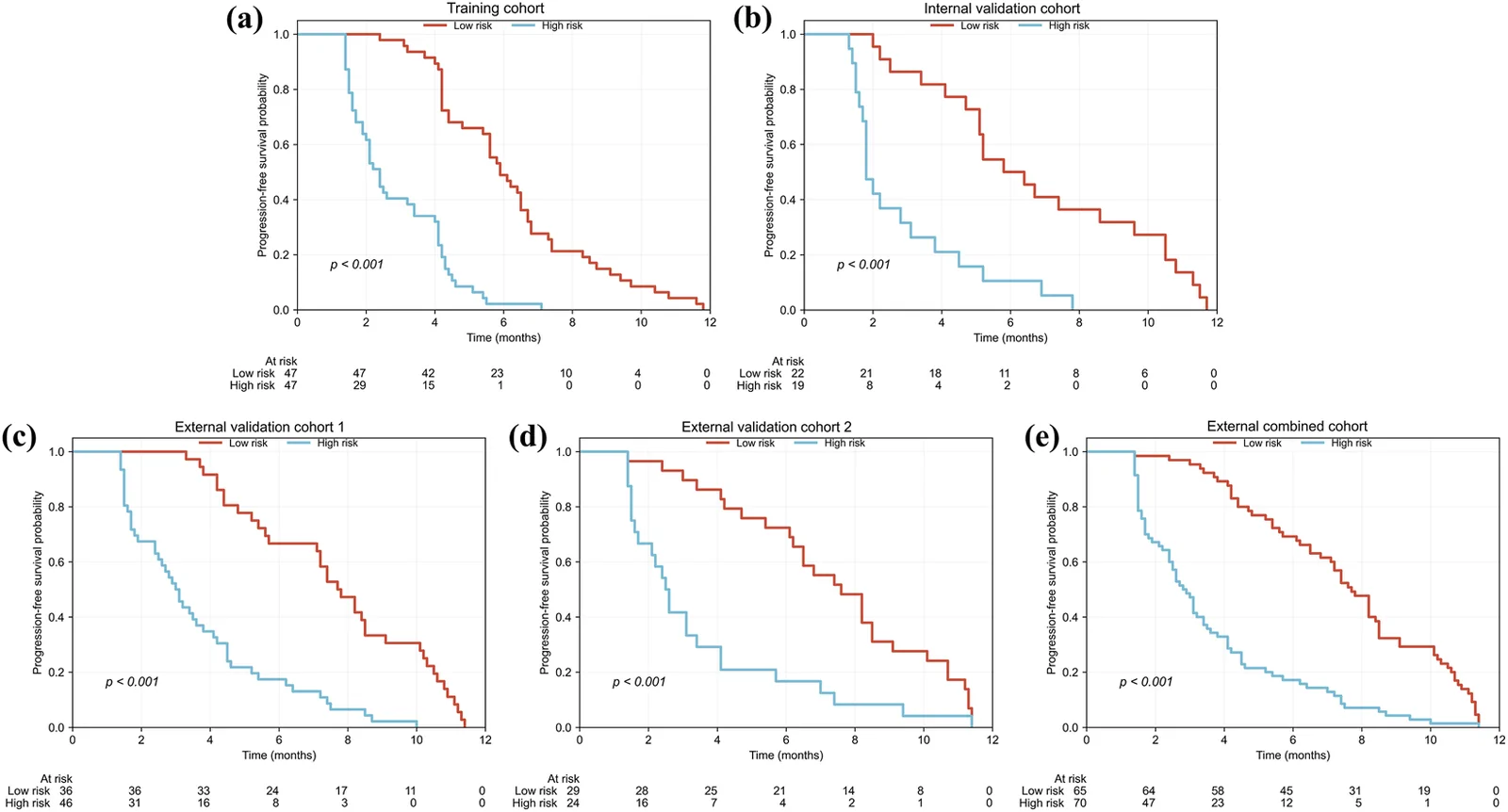
Kaplan–Meier survival analysis of the fusion model in each cohort. **(a–e)** Results for the training cohort, internal validation cohort, external test cohort 1, external test cohort 2, and external combined validation cohort, respectively. Progression-free survival differed significantly between the high-risk and low-risk groups stratified by the HPC risk score in all cohorts (all P < 0.001). HPC, habitat, peritumoral, and clinical features; PFS, progression-free survival.

In all five cohorts, patients in the high-risk group had significantly shorter PFS than those in the low-risk group, with all *P* < 0.001. In the training cohort, the median PFS was 2.40 months in the high-risk group and 5.90 months in the low-risk group (HR = 5.83, 95% CI: 3.55–9.58). In the internal validation cohort, the median PFS was 1.80 months and 5.80 months in the high-risk and low-risk groups, respectively (HR = 4.36, 95% CI: 2.13–8.92). In external Center 1, the median PFS was 3.00 months and 7.70 months in the high-risk and low-risk groups, respectively (HR = 4.34, 95% CI: 2.60–7.23). In external Center 2, the median PFS was 2.50 months and 7.60 months in the high-risk and low-risk groups, respectively (HR = 2.63, 95% CI: 1.50–4.61). In the external combined cohort, the median PFS was 2.80 months and 7.70 months in the high-risk and low-risk groups, respectively (HR = 3.27, 95% CI: 2.29–4.69) (Table 4). The confidence intervals of the hazard ratios in all cohorts did not cross 1, confirming the consistency and robustness of the HPC risk score in identifying high-risk patients.

**TABLE 4:** Kaplan–Meier analysis of risk stratification by the fusion model across cohorts.

Cohort	High-risk *n*	Low-risk *n*	Median PFS of high-risk group	Median PFS of low-risk group	HR (95% CI)	Log-rank *P*
Training cohort	47	47	2.40	5.90	5.829 (3.546–9.581)	<0.001
Internal validation cohort	19	22	1.80	5.80	4.361 (2.132–8.921)	<0.001
External validation cohort 1	46	36	3.00	7.70	4.339 (2.604–7.233)	<0.001
External validation cohort 2	24	29	2.50	7.60	2.632 (1.503–4.611)	<0.001
External combined cohort	70	65	2.80	7.70	3.274 (2.288–4.686)	<0.001

CI, confidence interval; HPC, habitat, peritumoral, and clinical features; HR, hazard ratio; PFS, progression-free survival.

Supplementary Table S3 showed that the clinical, habitat, peritumoral, and fusion models were all able to significantly stratify patients by risk across the different cohorts; however, the relative magnitudes of their HRs varied among the cohorts. The fusion model yielded the highest HR only in the training cohort, whereas the clinical, habitat, or peritumoral model had a higher HR in some validation cohorts. Nevertheless, the fusion model significantly distinguished between high- and low-risk patients in all validation cohorts, with HRs ranging from 2.63 to 4.34 and corresponding confidence intervals that did not cross 1, indicating a certain degree of stability in its risk-stratification performance. In addition, although the hazard ratio was relatively high in the training cohort (HR = 5.83), the fusion model maintained robust prognostic performance in the external validation cohorts (HR range: 2.63–4.34), and the corresponding confidence intervals did not cross 1, indicating stable prognostic stratification ability across different cohorts. In external Center 2, the hazard ratio of the fusion model was slightly attenuated (HR = 2.63), but remained highly statistically significant (*P* < 0.001).

### Nomogram construction and individualized prediction

A nomogram was constructed based on the HPC risk score (Figure 9), integrating the habitat feature score, peritumoral feature score, and AFP level to enable individualized prediction of 3- and 6-month PFS probabilities. Clinicians can estimate an individual patient’s risk of progression by assigning points to each variable and calculating the total score.

**FIGURE 9:**
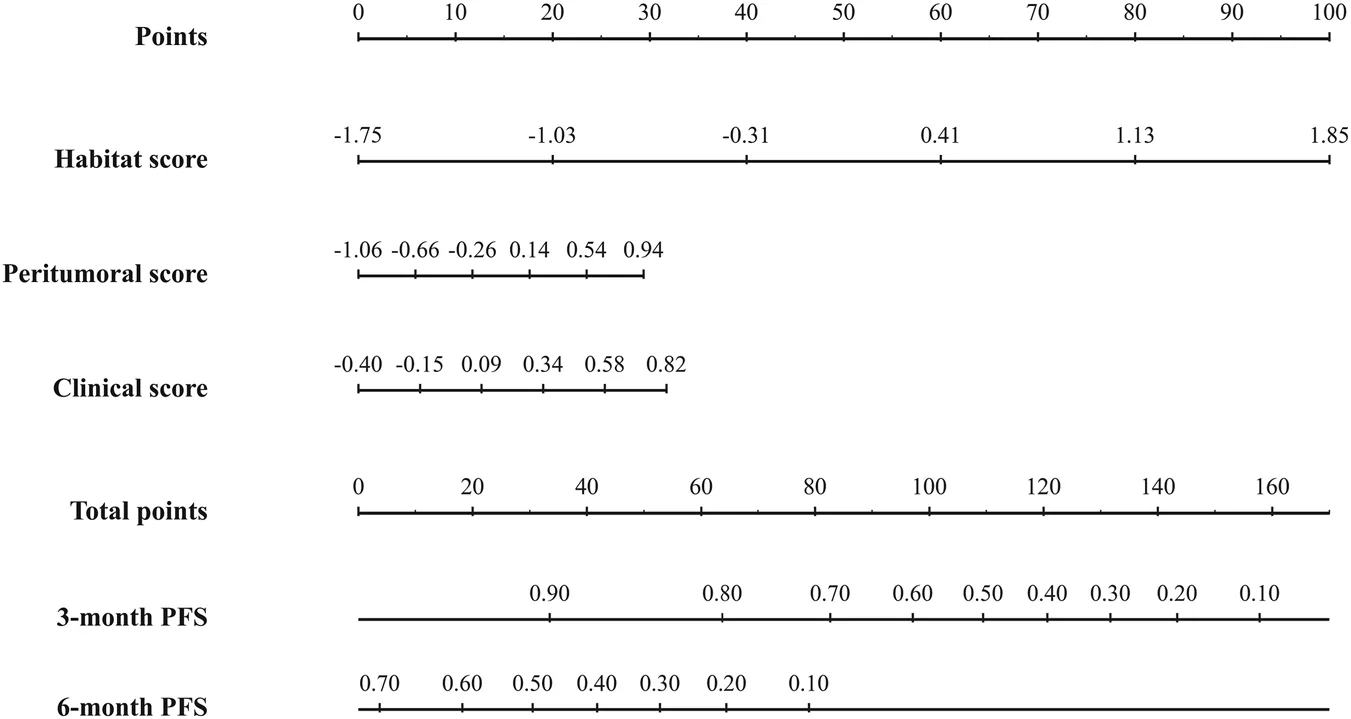
Nomogram for predicting progression-free survival based on the HPC risk score. HPC, habitat, peritumoral, and clinical features; PFS, progression-free survival.

## Discussion

Based on multicenter contrast-enhanced CT data, this study developed and validated a predictive model integrating tumor habitat radiomics, peritumoral radiomics features, and clinical variables to evaluate PFS after repeat TACE in patients with HCC, and further established an HPC risk score to enable individualized assessment of progression risk. For patients with HCC requiring multiple TACE sessions, early identification of those who may derive limited benefit from continued TACE remains a key issue in current clinical practice (Ashour et al., 2024). In this study, PFS after the second TACE session was used as the prediction endpoint, which more directly reflects the tumor-control effect of TACE and is less influenced by subsequent therapeutic interventions. This endpoint is therefore more closely aligned with clinical decision-making scenarios regarding whether to continue TACE or switch to alternative treatment strategies (Adhoute et al., 2015; Sieghart et al., 2013; Villani et al., 2021). Previous studies have also emphasized that Ogasawara et al., 2020, for patients with limited benefit from TACE, timely identification and appropriate adjustment of treatment strategies may help improve long-term prognosis. Therefore, the HPC risk score developed in this study may help identify patients at high risk of early progression, thereby providing evidence to support combination systemic therapy, treatment strategy switching, and intensified follow-up (Zhou et al., 2024).

It should be noted that previous retreatment scores, such as ART and ABCR, were not included as direct comparator models in this study. The main reason is that these scores are primarily used to evaluate overall survival or retreatment benefit after repeated TACE, whereas this study focused on PFS after the second TACE session. Therefore, their prediction targets and clinical application scenarios are not completely consistent. Directly evaluating the stratification ability of OS-oriented scores using PFS may lead to a methodologically unfair comparison. Accordingly, this study placed greater emphasis on comparing the incremental value of different information sources, namely, the performance differences among the clinical model, single radiomics models, and the fusion model.

Unlike traditional studies based on whole-tumor features, this study further focused on intratumoral spatial heterogeneity. Previous retreatment-related scoring systems, such as the ART score and ABCR score (Adhoute et al., 2015; Sieghart et al., 2013), were mainly constructed using limited clinical and imaging parameters and therefore may not fully reflect the highly heterogeneous biological characteristics of HCC. In recent years, although some studies have attempted to apply deep learning and radiomics to the prediction of TACE efficacy (Dai et al., 2025), most have remained focused on whole-tumor feature extraction and have lacked explicit modeling of intratumoral spatial architecture. In this study, the tumor was partitioned into distinct habitats through voxel-level clustering and combined with peritumoral regional features, thereby characterizing disease status from both intratumoral structural and peritumoral microenvironmental perspectives. Previous studies have shown that different intratumoral imaging phenotypic regions often correspond to distinct vascular supply patterns, degrees of necrosis, and cellular densities (Li et al., 2024). Meanwhile, the peritumoral region may contain imaging information related to the invasive tumor margin and microvascular invasion (Zou et al., 2025; Wang et al., 2024b). Both types of features demonstrated favorable prognostic value in this study, suggesting that intratumoral spatial heterogeneity and the peritumoral microenvironment may jointly influence TACE efficacy and progression risk. A 5-mm peritumoral region was selected in this study to balance the capture of local microenvironmental information immediately surrounding the tumor against interference from non-tumoral tissues. The liver parenchyma adjacent to the tumor margin may contain imaging information related to invasive growth, microvascular invasion, and alterations in the local microenvironment. A previous CT-based radiomics study of HCC compared 5-mm and 10-mm peritumoral regions and found that the 5-mm peritumoral region showed favorable performance in predicting microvascular invasion, with the model combining arterial-phase intratumoral features and 5-mm peritumoral features achieving the best predictive performance (Wang et al., 2024b). In contrast, further expansion of the peritumoral region may include more normal liver parenchyma, large vessels, and other adjacent structures, thereby diluting tumor-related local information and increasing noise in the radiomics features. Therefore, a 5-mm margin was considered a reasonable range that balanced biological relevance and regional specificity. Nevertheless, different peritumoral expansion ranges were not directly compared in this study. Future studies should systematically evaluate 3-mm, 5-mm, 10-mm, and other peritumoral ranges to determine the optimal region for predicting progression after the second TACE. From the perspective of imaging phenotype, the two habitat subregions identified in this study may correspond to different biological states within the tumor. After ranking according to the mean CT value in the portal venous phase, H1 appeared as a relatively hypoenhanced region, whereas H2 appeared as a more hyperenhanced region. Previous studies (Chartampilas et al., 2022; Agnello et al., 2025) have shown that different enhancement patterns within HCC are often associated with tumor vascular supply, microcirculatory characteristics, and histological heterogeneity. In combination with the findings of this study, hypoenhanced regions may more likely reflect hypoperfusion, hypoxia, or partial necrotic components, whereas hyperenhanced regions may indicate more abundant abnormal arterial supply and more active tumor biological behavior. Because TACE primarily exerts its therapeutic effect by blocking the tumor blood supply (Forner et al., 2018), different tumor regions may vary in their sensitivity to ischemia and chemotherapeutic agents. Particularly in patients undergoing repeat TACE, residual viable tissue often coexists with ischemic or necrotic regions within the tumor after one or more embolization procedures. Radiomics features extracted from the whole ROI are therefore susceptible to averaging effects, whereas habitat analysis can separately model distinct imaging phenotypes after partitioning them into different subregions. Thus, habitat analysis may more sensitively capture high-risk regional information associated with subsequent progression. This may partly explain the stable prognostic value demonstrated by habitat features in this study. However, the above interpretations are based primarily on indirect inferences from imaging phenotypes and previous studies. Because pathological tissue or molecular genomic data corresponding to the imaging time points were unavailable in this study, we were unable to directly validate the associations between the habitat subregions and hypoxia-related gene expression, microvessel density, angiogenesis-related molecules, immune cell infiltration, or the extent of tumor necrosis. Therefore, the true biological basis of the different habitat subregions requires further validation through imaging–pathology correlation analyses, perfusion imaging, or molecular biomarkers.

The fusion model demonstrated good predictive performance across all cohorts; however, its statistical advantage over the individual models was not entirely consistent. In particular, differences in the C-index and time-dependent AUC between the fusion model and the habitat or peritumoral model did not reach statistical significance in some validation cohorts. Therefore, the findings support the potential value of integrating multidimensional information but do not establish that the fusion model significantly outperformed every individual model across all cohorts. This suggests that lesion habitat and peritumoral features may capture partially overlapping prognostic information, reflecting the strong intrinsic association among imaging phenotypes related to intratumoral angiogenesis, hypoxia, and cellular proliferation. In other words, imaging-derived spatial heterogeneity information already played a dominant role in outcome prediction, whereas clinical variables contributed more to enhancing model robustness and generalizability. In this study, AFP was the only clinical variable ultimately incorporated into the fusion model. Although the coefficient of AFP was numerically small and its HR was close to 1.0, AFP was entered as a continuous variable in its original unit of ng/mL. Both estimates therefore represent the effect associated with a 1-ng/mL increase and should be interpreted in relation to the measurement scale of AFP. Because AFP levels may differ by hundreds or thousands of ng/mL between patients, a small per-unit effect does not necessarily indicate a negligible contribution to risk prediction. Meanwhile, AFP primarily reflects the overall tumor burden and biological aggressiveness of the tumor (Mehta et al., 2020), whereas habitat and peritumoral radiomics features further supplement information regarding intratumoral spatial heterogeneity and the status of the peritumoral microenvironment (Xia et al., 2023). The combination of these three components can therefore achieve more stable risk stratification.

The model calibration results showed that the predicted probabilities were generally consistent with the observed outcomes in most cohorts, although the model slightly underestimated the observed PFS probabilities in some external validation cohorts. From a clinical perspective, such slight underestimation is not entirely unacceptable. In settings with substantial uncertainty, moderately conservative predictions may be more helpful than overly optimistic risk estimates in prompting clinicians to adopt more cautious follow-up and treatment decisions. From a methodological perspective, this phenomenon may be related to temporal shifts between the training and validation cohorts. In recent years, with the optimization of TACE techniques and the gradual integration of targeted agents and immunotherapy into the HCC treatment paradigm, overall patient outcomes have improved compared with earlier periods (Ding and Li, 2025; Finn et al., 2020), which may have resulted in better actual prognoses than model-predicted outcomes in some validation cohorts. In addition, compared with 3-month PFS, calibration for 6-month and longer-term predictions slightly decreased, suggesting that subsequent treatments and dynamic tumor evolution factors not included in the model may gradually influence outcomes as the prediction horizon extends. Therefore, the stable performance of this model within the critical 3- to 6-month decision-making window may provide greater reference value for early clinical intervention. Decision curve analysis further validated the clinical utility of the model. Across all cohorts, the fusion model achieved higher net benefit over a broad range of threshold probabilities and consistently outperformed the “treat-all” and “treat-none” strategies. Its advantage was particularly evident in the intermediate threshold range, which corresponds to the most common zone of clinical decision-making uncertainty. In other words, in the gray zone of whether to continue TACE, the model can provide additional information to help reduce ineffective treatment while avoiding missed identification of patients who may still benefit. These findings suggest that the model not only has favorable predictive performance but also possesses potential for clinical translation. From the perspective of potential clinical application, the nomogram may be used to identify patients at high risk of early progression after the second TACE. For high-risk patients, the model results may prompt the clinical team to more cautiously assess the expected benefit of continuing repeat TACE alone and to consider earlier combination with targeted therapy or immunotherapy, as well as appropriately shortened imaging follow-up intervals. For low-risk patients, the model may provide supportive information for continuing the current treatment strategy and routine follow-up. However, this study did not compare the effectiveness of different treatment strategies or determine whether model-guided treatment modification could improve patient outcomes. Therefore, the nomogram is currently intended primarily for risk stratification and as an aid to clinical assessment; it cannot replace multidisciplinary clinical decision-making, and its clinical utility requires further validation in prospective studies.

In addition, during external validation, the model maintained relatively consistent performance across different centers, suggesting a certain degree of generalizability. In external Center 2, the hazard ratio of the fusion model was relatively lower than those in the other cohorts, but it remained statistically significant. This phenomenon may be related to differences in patient composition, tumor heterogeneity, scanning parameters, and treatment management strategies among different centers. Such performance fluctuations are relatively common in multicenter radiomics studies. However, in the external combined cohort, model performance became stable again, indicating that the model still had favorable generalizability across different centers, which is particularly critical for its clinical implementation.

This study still has several limitations. First, this study had a retrospective design, and although multicenter external validation was included, selection bias may still exist. The original multicenter research database did not contain structured records of the exact dates of the initial and second TACE procedures for all patients, and complete patient-level dates could not be reliably retrieved retrospectively across the three centers. Therefore, the mean or median interval between the two procedures could not be reliably calculated, and the potential effect of the treatment interval on PFS after the second TACE could not be evaluated. Moreover, the three participating centers did not prospectively apply uniform criteria for selecting patients for repeat TACE or a uniform definition of TACE refractoriness or failure. Differences in treatment decision-making among the centers may therefore have introduced additional selection bias. Second, differences in CT scanners, scanning parameters, and image segmentation across centers may affect the stability of radiomics features. Third, the initial and second TACE procedures may have differed in terms of embolic agent selection, embolic material dose, degree of catheter superselection, embolization endpoint, and operator experience. These technical treatment-related factors may have influenced local tumor control and PFS after the second TACE. Owing to the limited completeness of the retrospective multicenter data, these procedural parameters could not be uniformly quantified or incorporated into the multivariable analysis. Therefore, unmeasured technical treatment-related factors may have resulted in residual confounding, and the observed prognostic differences cannot be attributed entirely to imaging features and clinical variables. Fourth, this study developed the model using only baseline images acquired before the initial TACE and did not incorporate early imaging changes after the initial TACE or information on subsequent treatments. Treatments administered after the second TACE were not recorded, and differences in subsequent treatment, follow-up intervals, and imaging assessment may have affected the observed PFS. Future prospective studies should systematically collect and adjust for these factors using standardized imaging follow-up and response assessment. Although both arterial-phase and portal venous-phase images were used, the habitat subregions derived from a single baseline time point provided only a cross-sectional representation and may not fully capture the spatiotemporal heterogeneity of HCC. The extent and distribution of lipiodol deposition, residual tumor enhancement, and changes in tumor blood supply and the extent of necrosis after the initial TACE may provide additional information for predicting PFS after the second TACE. However, owing to the retrospective multicenter design, the timing and imaging protocols of follow-up examinations were not consistent across centers, precluding the reliable extraction of standardized delta-radiomics features (Huang et al., 2024). Future prospective studies may further improve the predictive performance of the model by integrating standardized multi-time-point imaging data. Fifth, pathological or genomic information was not integrated in this study, which limited the in-depth interpretation of the potential biological significance of habitat features. Sixth, owing to the limited overall sample size and the number of events in each validation cohort, this study was unable to conduct sufficiently robust and reliable subgroup analyses according to clinical characteristics such as AFP level, tumor burden, tumor number, BCLC stage, liver function status, and the presence or absence of portal vein tumor thrombosis. Therefore, the predictive stability and applicability of the model in specific clinical subgroups require further validation in larger prospective cohorts. In addition, ICC analysis of whole-tumor and peritumoral features reflected the reproducibility of manual segmentation but did not assess habitat clustering stability. Clustering stability across different random seeds or initialization conditions was not quantitatively evaluated using ARI or NMI, and therefore requires further validation.Finally, tumor segmentation still relied on manual delineation and therefore involved a certain degree of subjectivity. Future studies may incorporate automatic segmentation techniques, multi-time-point imaging data, and prospective multicenter validation to further improve model stability and clinical generalizability.

In summary, the fusion model developed in this study can comprehensively assess the risk of progression after repeat TACE from multiple perspectives, including intratumoral heterogeneity, the peritumoral microenvironment, and clinical status, and demonstrated favorable stability in multicenter data. This model may serve as an imaging-based tool to assist risk stratification and treatment strategy adjustment during repeat TACE, thereby supporting precision interventional treatment for patients with HCC.

## Data Availability

The data analyzed in this study is subject to the following licenses/restrictions: The datasets generated and/or analyzed during the current study are not publicly available due to patient privacy concerns and institutional data-sharing policies but may be obtained from the corresponding author upon reasonable request and with appropriate ethical approval. Requests to access these datasets should be directed to Liming Cui, limingcui1974@163.com.

## References

[B1] Adhoute X. Penaranda G. Naude S. Raoul J. L. Perrier H. Bayle O. (2015). Retreatment with TACE: the ABCR SCORE, an aid to the decision-making process. J. Hepatol. 62 (4), 855–862. 10.1016/j.jhep.2014.11.014 25463541

[B2] Agnello F. Taibbi A. Galia M. Orlando A. Gagliardo C. Bartolotta T. V. (2025). Hepatocellular carcinoma treatment response: imaging findings and criteria. World J. Radiol. 17 (10), 108804. 10.4329/wjr.v17.i10.108804 41180907 PMC12576714

[B3] Ashour R. Rewisha E. Rady M. A. Elkhadry S. W. Abdelhalim H. Atef M. (2024). Effectiveness of sorafenib in treating intermediate-stage hepatocellular carcinoma patients refractory to transarterial chemoembolization. BMC Cancer 24 (1), 1466. 10.1186/s12885-024-13199-1 39609726 PMC11603851

[B4] Cao J. Zhu T. Sun Q. Liu Y. Yu H. Guo X. (2026). MRI-based clinical-radiomics-habitat model for predicting prognosis of hepatocellular carcinoma patients treated with HAIC. Front. Oncol. 16, 1764150. 10.3389/fonc.2026.1764150 41684595 PMC12890658

[B5] Chartampilas E. Rafailidis V. Georgopoulou V. Kalarakis G. Hatzidakis A. Prassopoulos P. (2022). Current imaging diagnosis of hepatocellular carcinoma. Cancers 14 (16), 3997. 10.3390/cancers14163997 36010991 PMC9406360

[B6] Dai Y. Liu D. Xin Y. Li Y. Wang D. He B. (2023). Efficacy and interpretability analysis of noninvasive imaging based on computed tomography in patients with hepatocellular carcinoma after initial transarterial chemoembolization. Acad. Radiol. 30, S61–S72. 10.1016/j.acra.2023.05.027 37393179

[B7] Dai Y. Zhao S. Wu Q. Zhang J. Zeng X. Jiang H. (2025). A CT-Based deep learning radiomics scoring system for predicting the prognosis to repeat TACE in patients with hepatocellular carcinoma: a multicenter cohort study. J. Hepatocell. Carcinoma 12, 1647–1659. 10.2147/jhc.S525920 40756248 PMC12317699

[B8] Ding G. Li K. (2025). A CT-Based clinical-radiomics nomogram for predicting the overall survival to TACE combined with camrelizumab and apatinib in patients with advanced hepatocellular carcinoma. Acad. Radiol. 32 (4), 1993–2004. 10.1016/j.acra.2024.10.052 39578199

[B9] Filho A. M. Laversanne M. Ferlay J. Colombet M. Piñeros M. Znaor A. (2025). The GLOBOCAN 2022 cancer estimates: data sources, methods, and a snapshot of the cancer burden worldwide. Int. J. Cancer 156 (7), 1336–1346. 10.1002/ijc.35278 39688499

[B10] Finn R. S. Qin S. Ikeda M. Galle P. R. Ducreux M. Kim T. Y. (2020). Atezolizumab plus bevacizumab in unresectable hepatocellular carcinoma. N. Engl. J. Med. 382 (20), 1894–1905. 10.1056/NEJMoa1915745 32402160

[B11] Forner A. Reig M. Bruix J. (2018). Hepatocellular carcinomia. Lancet 391 (10127), 1301–1314. 10.1016/s0140-6736(18)30010-2 29307467

[B12] Heimbach J. K. Kulik L. M. Finn R. S. Sirlin C. B. Abecassis M. M. Roberts L. R. (2018). AASLD guidelines for the treatment of hepatocellular carcinoma. Hepatology 67 (1), 358–380. 10.1002/hep.29086 28130846

[B13] Huang Y. Qian H. (2024). Advancing hepatocellular carcinoma management through peritumoral radiomics: enhancing diagnosis, treatment, and prognosis. J. Hepatocell. Carcinoma 11, 2159–2168. 10.2147/jhc.S493227 39525830 PMC11546143

[B14] Huang W. Pan Y. Wang H. Jiang L. Liu Y. Wang S. (2024). Delta-radiomics analysis based on multi-phase contrast-enhanced MRI to predict early recurrence in hepatocellular carcinoma after percutaneous thermal ablation. Acad. Radiol. 31 (12), 4934–4945. 10.1016/j.acra.2024.06.002 38902111

[B15] Huang Z. Huang W. Jiang L. Zheng Y. Pan Y. Yan C. (2025). Decision fusion model for predicting microvascular invasion in hepatocellular carcinoma based on multi-MR habitat imaging and machine-learning classifiers. Acad. Radiol. 32 (4), 1971–1980. 10.1016/j.acra.2024.10.007 39472207

[B16] Isensee F. Jaeger P. F. Kohl S. A. A. Petersen J. Maier-Hein K. H. (2021). nnU-Net: a self-configuring method for deep learning-based biomedical image segmentation. Nat. Methods 18 (2), 203–211. 10.1038/s41592-020-01008-z 33288961

[B17] Lencioni R. Llovet J. M. (2010). Modified RECIST (mRECIST) assessment for hepatocellular carcinoma. Semin. Liver Dis. 30 (1), 52–60. 10.1055/s-0030-1247132 20175033 PMC12268942

[B18] Li N. Wan X. Zhang H. Zhang Z. Guo Y. Hong D. (2022). Tumor and peritumor radiomics analysis based on contrast-enhanced CT for predicting early and late recurrence of hepatocellular carcinoma after liver resection. BMC Cancer 22 (1), 664. 10.1186/s12885-022-09743-6 35715783 PMC9205126

[B19] Li S. Dai Y. Chen J. Yan F. Yang Y. (2024). MRI-based habitat imaging in cancer treatment: current technology, applications, and challenges. Cancer Imaging 24 (1), 107. 10.1186/s40644-024-00758-9 39148139 PMC11328409

[B20] Llovet J. M. Kelley R. K. Villanueva A. Singal A. G. Pikarsky E. Roayaie S. (2021). Hepatocellular carcinoma. Nat. Rev. Dis. Prim. 7 (1), 6. 10.1038/s41572-020-00240-3 33479224 PMC13537433

[B21] Mehta N. Dodge J. L. Grab J. D. Yao F. Y. (2020). National experience on down‐staging of hepatocellular carcinoma before liver transplant: influence of tumor burden, alpha‐fetoprotein, and wait time. Hepatology 71 (3), 943–954. 10.1002/hep.30879 31344273 PMC8722406

[B22] Ogasawara S. Ooka Y. Koroki K. Maruta S. Kanzaki H. Kanayama K. (2020). Switching to systemic therapy after locoregional treatment failure: definition and best timing. Clin. Mol. Hepatol. 26 (2), 155–162. 10.3350/cmh.2019.0021n 31937081 PMC7160341

[B23] Raoul J.-L. Gilabert M. Piana G. (2014). How to define transarterial chemoembolization failure or refractoriness: a European perspective. Liver Cancer 3 (2), 119–124. 10.1159/000343867 24945002 PMC4057788

[B24] Sieghart W. Hucke F. Pinter M. Graziadei I. Vogel W. Müller C. (2013). The ART of decision making: retreatment with transarterial chemoembolization in patients with hepatocellular carcinoma. Hepatology 57 (6), 2261–2273. 10.1002/hep.26256 23316013

[B25] Van Griethuysen J. J. Fedorov A. Parmar C. Hosny A. Aucoin N. Narayan V. (2017). Computational radiomics system to decode the radiographic phenotype. Cancer Res. 77 (21), e104–e107. 10.1158/0008-5472.CAN-17-0339 29092951 PMC5672828

[B26] Villani R. Cavallone F. Sangineto M. Fioravanti G. Romano A. D. Serviddio G. (2021). Management of intermediate-stage hepatocellular carcinoma in the elderly with transcatheter arterial chemoembolization failure: retreatment or switching to systemic therapy. Int. J. Clin. Pract. 75 (4), e13733. 10.1111/ijcp.13733 32981175

[B27] Wang Y. Li M. Zhang Z. Gao M. Zhao L. (2024a). Application of radiomics in the efficacy evaluation of transarterial chemoembolization for hepatocellular carcinoma: a systematic review and meta-analysis. Acad. Radiol. 31 (1), 273–285. 10.1016/j.acra.2023.08.001 37684182

[B28] Wang F. Cheng M. Du B. Li J. Li L. Huang W. (2024b). Predicting microvascular invasion in small (≤5 cm) hepatocellular carcinomas using radiomics-based peritumoral analysis. Insights Imaging 15 (1), 90. 10.1186/s13244-024-01649-0 38530498 PMC10965872

[B29] Xia T.-Y. Zhou Z.-H. Meng X.-P. Zha J.-H. Yu Q. Wang W.-L. (2023). Predicting microvascular invasion in hepatocellular carcinoma using CT-based radiomics model. Radiology 307 (4), e222729. 10.1148/radiol.222729 37097141

[B30] Zangos S. Eichler K. Balzer J. O. Straub R. Hammerstingl R. Herzog C. (2007). Large-sized hepatocellular carcinoma (HCC): a neoadjuvant treatment protocol with repetitive transarterial chemoembolization (TACE) before percutaneous MR-guided laser-induced thermotherapy (LITT). Eur. Radiol. 17 (2), 553–563. 10.1007/s00330-006-0343-x 16896704

[B31] Zhang S. Wang W.-S. Zhong B.-Y. Ni C.-F. (2022). Subsequent treatment after transarterial chemoembolization failure/refractoriness: a review based on published evidence. J. Clin. Transl. Hepatol. 10 (4), 740–747. 10.14218/JCTH.2021.00336 36062280 PMC9396332

[B32] Zhou C. Chang B. Xiang Z. Li Z. Wu C. Bai M. (2024). Transarterial chemoembolization (TACE) combined with lenvatinib *versus* TACE alone in intermediate-stage hepatocellular carcinoma patients beyond up-to-seven criteria: a retrospective, propensity score-matched analysis. Acad. Radiol. 31 (11), 4456–4465. 10.1016/j.acra.2024.04.045 38760273

[B33] Zou W. Gu M. Chen H. He R. Zhao X. Jia N. (2025). Radiomics with machine learning improves the prediction of microscopic peritumoral small cancer foci and early recurrence in hepatocellular carcinoma. Acad. Radiol. 32 (10), 5774–5788. 10.1016/j.acra.2025.07.018 40730778

[B34] Zwanenburg A. Vallières M. Abdalah M. A. Aerts H. J. Andrearczyk V. Apte A. (2020). The image biomarker standardization initiative: standardized quantitative radiomics for high-throughput image-based phenotyping. Radiology 295 (2), 328–338. 10.1148/radiol.2020191145 32154773 PMC7193906

