# Supplementary material for: CT habitat and peritumoral radiomics for predicting progression after repeat TACE in hepatocellular carcinoma: a multicenter study
Source: Front Pharmacol. 2026 Oct 14;17:1942002. doi: 10.3389/fphar.2026.1942002 (PMC13713701; doi:10.3389/fphar.2026.1942002)
Supplement: Supplementary file 1 [file Supplementaryfile1.docx]

**Supplementary Figure**

**Supplementary Figure S1.** LASSO-Cox regression analysis of radiomics features.

**Supplementary Figure S2**

**Supplementary Figure S3**

**Supplementary Figure S4**

**Supplementary Figure S5**

**Supplementary Figure S6**

**Supplementary Figure S7**.Brier score assessment of the fusion model.

**Supplementary Figure S1.** LASSO-Cox regression analysis of radiomics features.

(a) Cross-validation curve, with cross-validated partial likelihood deviance on the y-axis and λ on the x-axis; the left dashed line indicates the λ value with the minimum error (λ.min), and the right dashed line indicates the optimal λ value within 1 standard error (λ.1se). (b) LASSO coefficient profiles, in which each curve represents the trajectory of the regression coefficient for one radiomics feature as λ changes; the vertical dashed line indicates the optimal λ selected by five-fold cross-validation.
**Abbreviations:** LASSO, least absolute shrinkage and selection operator.

**Supplementary Figures S2-S6.** Pairwise comparisons of model performance across cohorts.

Pairwise comparisons were performed among the fusion, habitat, peritumoral, and clinical models in the training, internal validation, external validation 1, external validation 2, and combined external validation cohorts, respectively. (a) C-index comparisons; (b-d) DeLong tests for 3-, 6-, and 9-month time-dependent AUCs. Values indicate *P* values; *P* < 0.05 denotes statistical significance.
**Abbreviations:** AUC, area under the curve; PFS, progression-free survival.

**Supplementary Figure S7.** Brier score assessment of the fusion model.

(a) Time-dependent Brier score curves for each cohort, showing changes in prediction error over follow-up. (b) Bar plots of Brier scores at 3 and 6 months for each cohort; red and blue bars represent the 3-month and 6-month Brier scores, respectively.
**Abbreviations:** PFS, progression-free survival.

**Supplementary Table**

**Supplementary Table S1.** Final selected features and multivariate Cox regression results for each model.

| Model | Feature name | HR (95% CI) | *P* | Coefficient (Cox) | LASSO status |
| --- | --- | --- | --- | --- | --- |
| Clinical model | AFP | 1.001 (1.000–1.001) | <0.001 | 0.000613 | Not applicable |
| Habitat model | H1_V_original_firstorder_Entropy | 1.196 (0.924–1.548) | 0.175 | 0.178609 | Selected by LASSO |
|  | H1_V_original_firstorder_Minimum | 0.922 (0.700–1.213) | 0.560 | -0.081585 | Selected by LASSO |
|  | H1_V_wavelet-LHL_firstorder_Maximum | 1.342 (1.023–1.761) | 0.034 | 0.294339 | Selected by LASSO |
|  | H1_V_wavelet-HLL_gldm_LargeDependenceLowGrayLevelEmphasis | 0.928 (0.713–1.208) | 0.578 | -0.074730 | Selected by LASSO |
|  | H1_V_wavelet-HHL_glcm_Idmn | 1.177 (0.892–1.553) | 0.248 | 0.163260 | Selected by LASSO |
|  | H1_A_original_firstorder_Kurtosis | 1.198 (0.938–1.530) | 0.148 | 0.180728 | Selected by LASSO |
|  | H1_A_original_glcm_Idmn | 1.155 (0.857–1.557) | 0.345 | 0.143991 | Selected by LASSO |
|  | H1_A_original_gldm_LargeDependenceLowGrayLevelEmphasis | 0.927 (0.697–1.233) | 0.604 | -0.075440 | Selected by LASSO |
|  | H2_V_original_firstorder_Entropy | 1.314 (1.003–1.721) | 0.048 | 0.272806 | Selected by LASSO |
|  | H2_V_logarithm_glszm_LargeAreaEmphasis | 1.409 (1.110–1.788) | 0.005 | 0.342900 | Selected by LASSO |
|  | H2_A_wavelet-HLH_glszm_SmallAreaEmphasis | 1.110 (0.868–1.419) | 0.404 | 0.104593 | Selected by LASSO |
| Peritumoral model | V_original_shape_LeastAxisLength | 1.421 (1.208–1.672) | <0.001 | 0.351680 | Selected by LASSO |
|  | A_square_glszm_SizeZoneNonUniformityNormalized | 0.804 (0.649–0.997) | 0.047 | -0.218232 | Selected by LASSO |
|  | A_wavelet-HHH_firstorder_Median | 1.379 (1.123–1.694) | 0.002 | 0.321436 | Selected by LASSO |
| Fusion model | habitat_score | 1.342 (1.157–1.557) | <0.001 | 0.294300 | Not applicable |
|  | peri_score | 1.451 (1.138–1.849) | 0.003 | 0.371921 | Not applicable |
|  | AFP | 1.000 (1.000–1.000) | 0.012 | 0.000205 | Not applicable |

**Supplementary Table S2.** Comparison of risk stratification performance across models in all cohorts.

| Cohort | Model | HR (95% CI) | Log-rank *P* |
| --- | --- | --- | --- |
| Training Cohort | Clinical | 3.332 (2.106–5.272) | <0.001 |
|  | Habitat | 5.296 (3.174–8.838) | <0.001 |
|  | Peritumoral | 3.102 (1.979–4.862) | <0.001 |
|  | **Fusion** | **5.829 (3.546–9.581)** | **<0.001** |
| Internal Validation Cohort | Clinical | 4.941 (2.194–11.126) | <0.001 |
|  | Habitat | 4.626 (2.263–9.456) | <0.001 |
|  | Peritumoral | 3.599 (1.766–7.336) | <0.001 |
|  | **Fusion** | **4.361 (2.132–8.921)** | **<0.001** |
| External Validation Cohort 1 | Clinical | 3.500 (2.125–5.764) | <0.001 |
|  | Habitat | 4.667 (2.742–7.942) | <0.001 |
|  | Peritumoral | 4.871 (2.921–8.122) | <0.001 |
|  | **Fusion** | **4.339 (2.604–7.233)** | **<0.001** |
| External Validation Cohort 2 | Clinical | 3.163 (1.706–5.864) | <0.001 |
|  | Habitat | 3.155 (1.771–5.619) | <0.001 |
|  | Peritumoral | 2.120 (1.206–3.725) | 0.005 |
|  | **Fusion** | **2.632 (1.503–4.611)** | **<0.001** |
| External Combined Cohort | Clinical | 3.424 (2.324–5.046) | <0.001 |
|  | Habitat | 3.627 (2.511–5.239) | <0.001 |
|  | Peritumoral | 3.075 (2.145–4.409) | <0.001 |
|  | **Fusion** | **3.274 (2.288–4.686)** | **<0.001** |

**Supplementary Table S1.** Final selected features and multivariate Cox regression results for each model.

Features in the habitat and peritumoral models were selected using LASSO-Cox regression. Variables in the fusion model were the risk scores of each submodel, with final coefficients determined by multivariate Cox regression. In the LASSO status column, “Selected by LASSO” indicates that the feature retained a nonzero coefficient after LASSO selection, whereas “Not applicable” indicates that the variable was directly entered into the model as a precomputed score and did not undergo LASSO selection.
**Abbreviations:** A, arterial phase; CI, confidence interval; H1, Habitat 1; H2, Habitat 2; HR, hazard ratio; LASSO, least absolute shrinkage and selection operator; V, venous phase.

**Supplementary Table S2.** Comparison of risk stratification performance across models in all cohorts.

Except for the peritumoral model in external test cohort 2 (*P* = 0.005), all models showed log-rank P < 0.001 across all cohorts.
**Abbreviations:** CI, confidence interval; HR, hazard ratio.
